# High-accuracy hyperspectro-polarimetric real-time imaging via a deep learning empowered infrared meta-sensor

**DOI:** 10.1038/s41377-026-02407-1

**Published:** 2026-08-20

**Authors:** Huiming Luo, Jie Deng, Jing Zhou, Tianyuan Cui, Yuejie Zhang, Junwei Huang, Liwen Fu, Yonghao Bu, Ruowen Wang, Mengdie Shi, Xiaofei He, Xueling Guo, Jun Ning, Xiaoshuang Chen, Wei Lu

**Affiliations:** 1 State Key Laboratory of Infrared Physics, Shanghai Institute of Technical Physics, Chinese Academy of Sciences, Shanghai, 200083 China; 2 School of Physics and Optoelectronic Engineering, Hangzhou Institute for Advanced Study, University of Chinese Academy of Sciences, Hangzhou, 310024 China; 3 University of Chinese Academy of Sciences, Beijing, 101408 China; 4 Shanghai Key Laboratory of Optical Coatings and Spectral Modulation, Shanghai Institute of Technical Physics, Chinese Academy of Sciences, Shanghai, 200083 China; 5 School of Physical Science and Technology, ShanghaiTech University, Shanghai, 201210 China; 6 Suzhou Laboratory, Suzhou, 215000 Jiangsu China

**Keywords:** Metamaterials, Optoelectronic devices and components

## Abstract

Light inherently carries multidimensional information, including intensity, polarization, and spectrum. Employing a miniaturized device to simultaneously, instantaneously, and accurately capture the multidimensional information of incident light in a single exposure holds significant applications across numerous fields, but remains challenging. Here, we demonstrate high-accuracy hyperspectro-full-Stokes-polarimetric real-time imaging across a broad wavelength range (1150–1650 nm) with 167 spectral channels, via a metasurface integrated near infrared camera empowered by a residual attention network. The metasurface, which simultaneously performs polarization multiplexing and spectral dispersion, functions as a multidimensional encoder. A tailored deep learning network with a residual attention module is established and trained to reconstruct the multidimensional information of incident light with high accuracy. The key performance metrics—including spectral and image resolution, as well as spectral and polarization reconstruction accuracy—all surpass the best-reported specifications of hyperspectro-polarimetric cameras, with enhancements ranging from several-fold to one order of magnitude. Based on this approach, even intricately coupled multidimensional information can be resolved. In addition, the acquisition, processing, and inference time only takes 18 ms, allowing high-dimensional hyperspectral-polarimetric imaging at 55 frames per second in real time. This work offers a promising solution enabling high-accuracy hyperspectro-polarimetric real-time imaging with a compact camera.

## Abstract (web summary)

A metasurface-encoded infrared sensor with a deep-learning decoder enables single-shot real-time hyperspectro-polarimetric imaging.

## Graphical abstract

**Figure:**
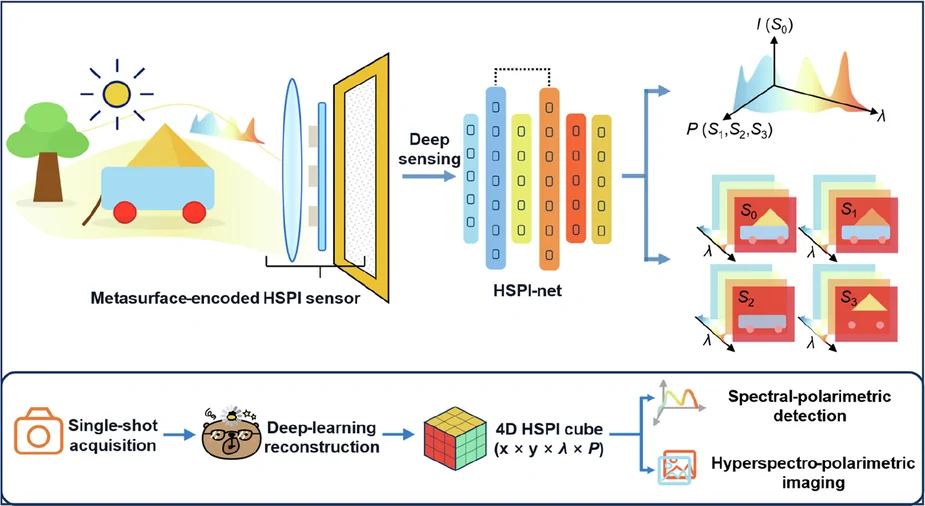

## Introduction

Light carries rich information across multiple dimensions, including intensity, polarization, and spectrum. Capturing multidimensional information of light allows for a more comprehensive characterization of targets, which is essential in applications such as optical communications^1,2^, remote sensing^3,4^, and bio-diagnostics^5,6^. In particular, for camouflaged or concealed targets designed through the regulation of radiative properties^7–12^, simultaneously acquiring both spectral and polarization characteristics is important for high-accuracy identification. Conventional approaches to achieving high-accuracy multidimensional optical sensing typically rely on bulky instrumentation (such as benchtop spectrometers), or require sequential acquisition strategies (such as spatial-division polarization measurements)^13^. Therefore, there is a long-standing demand for compact systems capable of real-time and single-shot acquisition of the multidimensional information carried by light^14–18^

With the rapid advancement in nanophotonics and nanotechnology, metasurface with unprecedented light manipulation capabilities have emerged as promising candidates to replace conventional optical components, or even entire systems. Despite significant progress, most existing studies only demonstrate sensing in only a few dimensions, such as spectral imaging^19–21^, polarimetric imaging^14,22–33^, or spectro-polarimetric detection without imaging^34–39^. Some works have reported polarimetric imaging at discrete wavelengths^40,41^. Other approaches have integrated micro-filter arrays with rotating polarization optics or metasurfaces to realize spectro-polarimetric imaging^42,43^.

More recently, compact devices capable of hyperspectro-polarimetric imaging (HSPI) have been first demonstrated in the visible-to-near-infrared range (400–900 nm). The device consists of a quarter-wave plate supported dispersive film and a microlens array^44^. Subsequently, a more compact hyperspectro-polarimetric camera, integrating an encoding metasurface with a CMOS image sensor, enabling real-time HSPI with a high spectral sensitivity across a broad wavelength range (700–1150 nm)^45^. In a closely related direction, a monolithic meta-grating-lens polarization camera^46^ has been demonstrated, in which polarization analysis, beam splitting, and imaging are integrated within a single metasurface layer, enabling real-time full-Stokes polarization imaging with a 14° field of view in the near-infrared.

These pioneering works demonstrate the feasibility of real-time, single-shot HSPI using compact devices and sparked broad research interest in this area, but the accuracy of the reconstructed multidimensional information is inadequate for practical applications.

In this work, we demonstrate deep-learning-empowered, high-accuracy, and real-time HSPI across a broad wavelength range (1150–1650 nm) with 167 spectral channels, via a metasurface integrated near infrared camera empowered by a residual attention network. The metasurface, composed of silicon nanopillars, simultaneously performs polarization multiplexing and spectral dispersion. Multidimensional infrared information (including spatially resolved intensity, polarization, and spectral composition) is encoded by the metasurface and captured by an InGaAs focal plane array (FPA). We design and implement a hyperspectral-polarization imaging residual attention network (HSPI-net), in which the residual structure stabilizes deep network training and the attention module adaptively enhances informative spectral-polarization features while suppressing redundancy, thereby improving feature representation. After training on a sufficiently large experimental dataset, we achieve high-accuracy, real-time HSPI. The spectral resolution (*λ*/Δ*λ* = 433) and image resolution of our device are twofold and sevenfold times higher than the best-reported value of previous HSPI devices, respectively. The spectrum reconstruction error (root-mean-square error (RMSE) 0.41%) and the polarization reconstruction error (angular deviation between the input and reconstructed Stokes vectors is 0.134°) of our device are 4.5-fold and more than one order of magnitude lower than the best-reported values. In addition, the acquisition, processing, and inference time only takes 18 ms, allowing high-dimensional hyperspectral-polarimetric imaging at 55 frames per second in real time. Our work develops a data-driven approach that paves the way for advanced multidimensional infrared sensing and holds great potential for applications in target recognition, machine vision, and beyond.

## Results

### Device structure and working principle

**Fig. 1:**
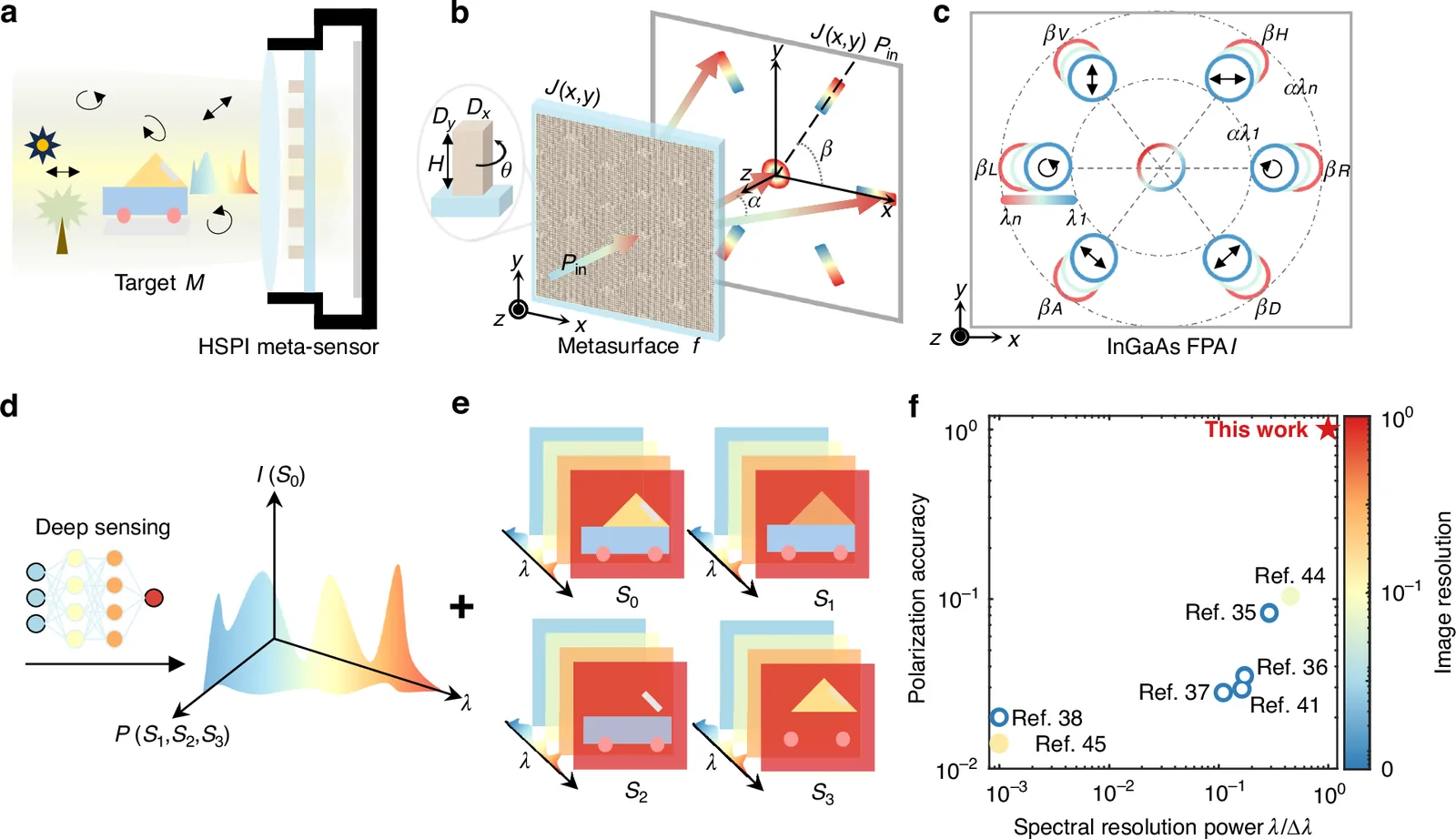
**Device structure and working principle.** **a** Schematic of the metasurface-empowered InGaAs FPA for deep-learning-empowered hyperspectral-polarimetric imaging. **b** The metasurface is illuminated by incident light (*P*_*in*_) with an unknown polarization state and spectral. The transmitted light (*J*(*x,y*)*P*_*in*_) is modulated by the metasurface’s spatially varying Jones matrix *J*(*x,y*). Inset: A single nanopillar unit within the metasurface, characterized by lateral dimensions *D*_*x*_, *D*_*y*_, and in-plane rotation angle *θ*, with a fixed height *H*. **c** Schematic of the encoded image captured by the InGaAs FPA. *α* and *β* represent the polar angle and azimuth angle in the polar coordinate system for six channels. Schematic representation of multidimensional optical information **d** as a high-dimensional parameter space, and **e** as a hyperspectro-polarimetric data cube consisting of spatially resolved intensity, polarization, and spectral information. **f** Comparison of polarization reconstruction error, spectral resolution power, and image resolution between our device and previously reported HSP/HSPI devices. (Solid/hollow markers indicate the presence/absence of each imaging capability, and the color represents the image resolution magnitude)

The multidimensional infrared information, including the spatially resolved polarization and spectral composition, is encoded by a metasurface and captured as a grayscale image by an InGaAs FPA (Fig. 1a). The metasurface is composed of silicon nanopillars, each defined by lateral dimensions *D*_*x*_, *D*_*y*_, and in-plane rotation angle *θ*, with a fixed height *H* = 1000 nm (Inset in Fig. 1b). Each nanopillar functions as an artificial birefringence meta-atom, engineered to independently manipulate a pair of orthogonal polarization states through a combination of accumulative and geometric phase control.

To enable full-Stokes polarization encoding, three types of unit cells are designed, each multiplexing two polarization channels: (1): horizontally linear polarization (H, 0°) and vertically linear polarization (V, 90°), (2): left-handed circular polarization (L) and right-handed circular polarization (R), (3): anti-diagonal linear polarization (A, 135°) and diagonal linear polarization (D, 45°). The unit cells are periodically arranged, with the nanopillar dimensions systematically varied to introduce the desired phase retardation between the *x*- and *y*-polarized components at each position. As a result, incident light is deflected into six distinct directions, each characterized by a pair of polar angle and azimuth angle in the polar coordinate system (*α*_*n*_, *β*_*n*_). The six designed channels correspond to (7.62°, 60°), (7.62°,120°), (7.62°, 240°), (7.62°, 300°), (8.12°, 180°) and (8.12°, 0°), (Fig. 1b, c). In parallel, the metasurface encodes spectral information through the structural dispersion of the nanopillars, radially separating different wavelengths in ascending order as the polar angle *α*_*n*_ increases for each channel. This dual encoding approach allows simultaneous spatial separation of polarization and spectral components over the FPA.

The incident light undergoes polarization-spectral modulation when penetrating the metasurface, and then forms a corresponding grayscale image on the InGaAs FPA. The HSPI, which can be represented by either a high-dimensional parametric space or a full-Stokes hyperspectral image data cube (Fig. 1d, e), is reconstructed from the grayscale imaging with the help of the HSPI network. In order to obtain high-throughput and high-quality data with corresponding ground-truth labels, we establish an optical setup with programmable and automated control of output wavelength and polarization state. Especially, we construct a double-path setup to generate spectral-polarization intricately coupled light. After training, the network enables accurate reconstruction of full-Stokes spectra, achieving real-time HSPI. The performance comparison in terms of polarization reconstruction error, spectral resolution, and imaging capability is shown in Fig. 1f (see SI Note 1 for details).

### Polarization and spectral encoding by the metasurface

The mechanisms of polarization and spectral encoding are detailed in this section. As shown in Fig. 1b, in order to transmit incident light with different polarization states into different channels, the phase profile of the metasurface is engineered based on the generalized Snell’s law^47,48^:

1 $${\varphi }_{n}(x,y)=\frac{2\pi }{{\lambda }_{d}}xsin({\alpha }_{n})cos({\beta }_{n})+\frac{2\pi }{{\lambda }_{d}}ysin({\alpha }_{n})sin({\beta }_{n})$$

where ${\varphi }_{n}(x,y)$ denotes the phase imparted to the transmitted light at the position (x,y) of the metasurface. Subscript *n* = H, V, A, D, L, or R indicates the polarization state of the incident light and corresponds to the six channels. *λ*_*d*_ denotes the designed wavelength of 1300 nm, *α*_*n*_ and *β*_*n*_ represent the polar angle and azimuth angle in the polar coordinate system. The angles (*α*_*n*_, *β*_*n*_) for the six channels are designed as (7.62°, 60°), (7.62°,120°), (7.62°, 240°), (7.62°, 300°), (8.12°, 180°), and (8.12°, 0°). The desired phase distribution is achieved by carefully engineering the spatial arrangement of diverse silicon nanopillars. The key structural parameters that govern the metasurface response (${\varphi }_{n}(x,y)$) include the pitch size (center-to-center distance between two neighboring nanopillars in the *x* or *y* direction), the height of the nanopillars *H*, the lateral dimensions of each nanopillar ${D}_{u}(x,y)$, ${D}_{v}(x,y)$, and the in-plane orientation angle of each nanopillar θ(x,y). In our design, the pitch size is fixed at 560 nm, and the height is fixed at 1000 nm. Each nanopillar imposes different phase shifts (i.e., ${\varPhi }_{u}(x,y)$ and ${\varPhi }_{v}(x,y)$) for light polarized along the two local orthogonal axes (*u*-axis and *v*-axis), enabling independent control of two orthogonal polarization components. These phase shifts (${\varPhi }_{u}(x,y)$, ${\varPhi }_{v}(x,y)$) are mainly controlled by the lateral dimensions (${D}_{u}(x,y)$,${D}_{v}(x,y)$).

For circularly or elliptically polarized light, the in-plane rotation of the nanopillars introduces a geometric phase component in addition to the propagation phase. Then, each nanopillar can be described by a Jones matrix^49^:

2 $$J(x,y)=R(-\theta (x,y))\cdot \left[\begin{array}{cc}{T}_{u}(x,y)\exp (i{\Phi }_{u}(x,y)) & \,0\\ 0\, & {T}_{v}(x,y)\exp (i{\Phi }_{v}(x,y))\end{array}\right]\cdot R(\theta (x,y))$$

Here, R(−θ(x,y)) and R(θ(x,y)) are two 2 × 2 rotation matrices that relate the global coordinate system (*x*-*y*) to the local coordinate system (*u*-*v*). ${T}_{u}(x,y)\exp (i{\varPhi }_{u}(x,y))$ and ${T}_{v}(x,y)\exp (i{\varPhi }_{v}(x,y))$ denote the complex transmission coefficients for the *u*- and *v*-polarized components, respectively. These coefficients are obtained through full-wave numerical simulation for each nanopillar geometry (details in “Methods”). Based on these simulations, two-dimensional contour maps of the transmission amplitude and phase as functions of the lateral dimensions (*D*_*u*_ and *D*_*v*_) are generated for both *u*- and *v*-polarization (see SI Note 2 for details).

As we design the nanopillars that the transmission amplitudes of the two orthogonal local polarizations are equal, i.e., ${T}_{u}(x,y)={T}_{v}(x,y)=T$, the Jones matrix further simplifies to:

3 $$J(x,y)=T\exp \left(i\frac{{\Phi }_{u}+{\Phi }_{v}}{2}\right)\left[\begin{array}{cc}\cos (\frac{\delta }{2})+i\,\cos (2\theta )\sin (\frac{\delta }{2}) & -i\,\sin (2\theta )\sin (\frac{\delta }{2})\\ -i\,\sin (2\theta )\sin (\frac{\delta }{2}) & \cos (\frac{\delta }{2})-i\,\cos (2\theta )\sin (\frac{\delta }{2})\end{array}\right]$$

where $\delta ={\varPhi }_{u}(x,y)-{\varPhi }_{v}(x,y)$ represents the phase retardation between the *E*_*u*_ and *E*_*v*_ components. The Jones matrix for different polarized vectors is derived in detail at Note 3.

**Fig. 2:**
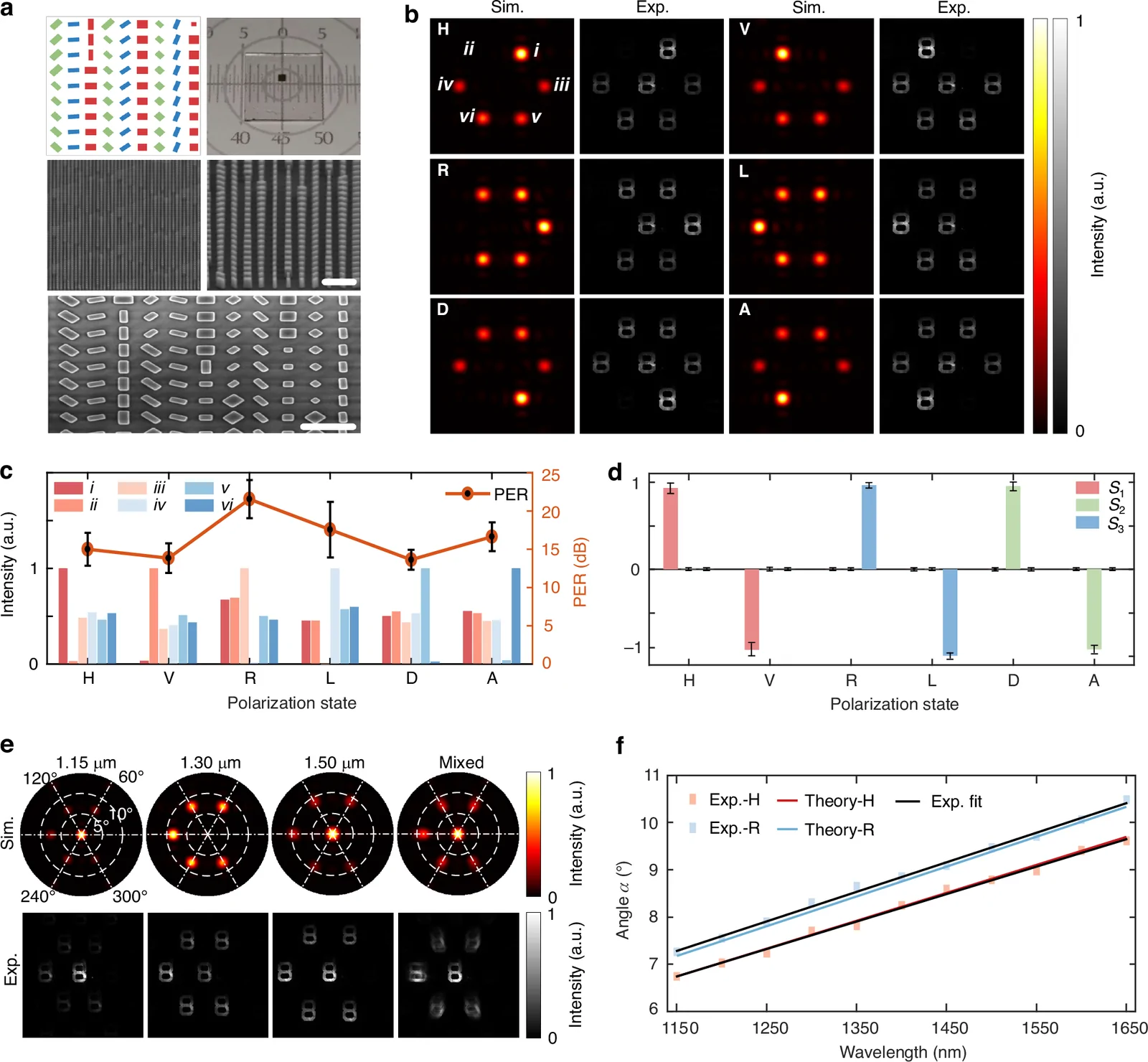
**Simulation and experimental characterization of the metasurface sample.** **a** Schematic diagram, optical microscope image, and scanning electron microscope (SEM) images of the fabricated metasurface sample. Side and top-view SEM images with scale bars of 1 μm in both views. **b** Theoretically simulated and experimentally measured far-field intensity distribution of the designed metasurface under 1300 nm incident light, showing six polarization channels. Inset H-A corresponds to different input polarization states. H: horizontal linear polarization (0°), V: vertical linear polarization (90°), L: left-handed circular polarization, R: right-handed circular polarization, A: anti-diagonal linear polarization (135°), and D: diagonal linear polarization (45°). **c** Normalized intensity of experimental distribution for different polarization channels in (**b**). Bars: normalized intensity in the six polarization channels (i–vi). Solid dots: mean polarization extinction ratio (PER) of the six channels under different incident polarization states, error bars represent the statistical standard deviations over about 3000 pixels. **d** Calculated Stokes parameters (*S*_1_, *S*_2_, *S*_3_) based on experimental data, the Stokes parameters are averaged over about 3000 pixels, and error bars represent the statistical standard deviations. **e** Theoretically simulated and experimentally measured far-field intensity distributions under left-handed circular polarization (LCP) illumination. Columns 1–3 correspond to wavelengths of 1150, 1300, and 1500 nm, while column 4 shows the results under simultaneous illumination with all three wavelengths. **f** Theoretically calculated and experimentally measured *α*_*n*_ of the vertical linear polarization (channel ii) and right-handed circular polarization (channel iii) at different incident wavelengths

Our metasurface is composed of three mutually nested sub-metasurfaces, as shown in Fig. 2a, first row. The sub-metasurface in red is used to differentiate the H and V polarization states; that in green is used to differentiate the A and D polarization states; and that in blue is used to differentiate the L and R polarization states. The metasurface was made of a silicon film (1000 nm thick) on a sapphire substrate, with a total patterned area of 960 × 960 μm^2^, nanopillars are arranged in a square lattice with a pitch size of 560 nm (Fig. 2a). Scanning electron microscope (SEM) images of the fabricated sample are shown in Fig. 2a, including both top and side views, clearly illustrating the uniformity and structural integrity of the nanopillars

For the H-V sub-metasurface, the rotation angle *θ* is fixed at 0° for all the nanopillars, and thus the *u*-*v* coordinate system is consistent with the *x*-*y* coordinate system. The lateral sizes (${D}_{u}(x,y)$ and ${D}_{v}(x,y)$) of each nanopillar are determined by minimizing the difference between the ideal complex transmission coefficients (i.e., $\exp (i{\varphi }_{H}(x,y))$ and $\exp (i{\varphi }_{V}(x,y))$) and the complex transmission coefficients at the nanopillar under *u*- and *v*-polarization illumination, respectively, based on the simulated transmission phase and amplitude as functions of *D*_*u*_ and *D*_*v*_ (SI Note 3). For the A-D sub-metasurface, the rotation angle *θ* is fixed at 45° for all the nanopillars. The lateral sizes of each nanopillar are determined by minimizing the difference between the ideal complex transmission coefficients (i.e., $\exp (i{\varphi }_{A}(x,y))$ and $\exp (i{\varphi }_{D}(x,y))$) and the complex transmission coefficients at the nanopillar under 45°- and 135°-polarization illumination, respectively. For the L-R sub-metasurface, the phase gradient is mainly controlled by the geometric phase that is associated with the rotation angle *θ*. The sub-metasurface converts the incident LCP (or RCP) light into RCP (or LCP) light while imposing the geometric phase on it. The *D*_*u*_ and *D*_*v*_ control the polarization conversion efficiency. The polarization converted part is deflected in the direction of $({\alpha }_{L},{\beta }_{L})$ or $({\alpha }_{R},{\beta }_{R})$, while the polarization unconverted part propagates in its original direction. Thus, the *D*_*u*_ and *D*_*v*_ are determined by maximizing the polarization conversion efficiency based on Eq. 3.

As shown in Fig. 2b, both theoretically simulated (spot patterns) and experimentally measured (“8”-shape mask) far-field intensity distributions are presented for different incident polarization states at a wavelength of 1300 nm. Subpanels (i–vi) correspond to incident polarizations H, V, D, A, L, R, respectively. The observed central light leakage originates from fabrication imperfections of the metasurface (see SI Note 7 for details). To quantitatively evaluate channel selectivity, we define the polarization extinction ratio (PER) as the ratio of orthogonal polarization intensities: ${\rm{PER}}=10\,\log ({I}_{n}/{I}_{o})$, where *I*_*n*_ is the intensity for the target polarization and *I*_*o*_ is for the its orthogonal counterpart. For the “8”-shaped mask imaging, the intensities *I*_*n*_ and *I*_*o*_ were integrated over approximately 3000 pixels per channel for quantitative analysis. The Stokes parameters are then calculated as: ${S}_{0}={I}_{H}+{I}_{V}$, ${S}_{1}={I}_{H}-{I}_{V}$, ${S}_{2}={I}_{D}-{I}_{A}$, ${S}_{3}={I}_{L}-{I}_{R}$, where *I*_*H*_, *I*_*V*_, *I*_*A*_, *I*_*D*_, *I*_*R*_ and *I*_*L*_ represent the integrated intensities of the six polarization channels, respectively. The normalized experimental intensities and PER values for all six polarization channels are summarized in Fig. 2c, the average PER is 16.38 dB. Based on the extracted intensities and the definition of Stokes parameters, the incident polarization states are reconstructed, as illustrated in Fig. 2d.

The spectral encoding is mainly governed by the structural dispersion of the nanopillar. The dispersive behavior can be described using the generalized Snell’s laws of refraction^47^. The phase gradient ($\nabla \varphi$) across the *x*-*y* plane of the metasurface (Eq. 1) is a function of the in-plane coordinates *x* and *y*. In other words, at each position (*x*, *y*), the metasurface deflects a portion of the normally incident light by an angle *α*^50^:

4 $${\left\{\begin{array}{c}sin({\alpha }_{\lambda {\rm{n}}})=\frac{\lambda }{2\pi }\sqrt{{\left(\frac{\partial {\varphi }_{n}(x,y)}{\partial x}\right)}^{2}+{\left(\frac{\partial {\varphi }_{n}(x,y)}{\partial y}\right)}^{2}}\\ tan({\beta }_{\lambda {\rm{n}}})=\,tan({\beta }_{n})\end{array}\right.}$$

Here, ${\alpha }_{\lambda {\rm{n}}}$ and ${\beta }_{\lambda {\rm{n}}}$ denote the polar and azimuth angle in the polar coordinate system for incident light of wavelength *λ* (*n* = H, V, A, D, L or R). By substituting Eq. 1 into Eq. 4, the variation of the deflection angle (*α*) induced by a change in the incident wavelength can be obtained. Thus, for each channel, a series of images is formed along the radial direction.

Figure 2e, Columns 1–3, displays the theoretically simulated (spot patterns) and experimentally measured (“8”-shape mask) far-field intensity distributions under LCP illumination with a wavelength of 1150, 1300, 1500 nm, respectively. The fourth column shows the results under simultaneous illumination with all three wavelengths. As the wavelength increases, the diffracted intensity patterns exhibit distinct radial separation, highlighting the wavelength-dependent dispersion capability of the metasurface. Figure 2f presents both the calculated and experimentally measured *α*_*n*_ across 1150–1650 nm, yielding an angular dispersion rate of 0.0063°/nm for circular polarization and 0.0058°/nm for linear polarization. The consistent radial dispersion observed across all polarization channels further validates the ability of the metasurface to project different wavelengths onto different positions on the FPA.

### Reconstruction performance for polarization and spectra information

The grayscale image on the InGaAs FPA can be expressed as a two-dimensional intensity array:

5 $${I}_{x,y}=\sum _{x\mathrm{'},y\mathrm{'},S,\lambda }{f}_{x,y,x\mathrm{'},y\mathrm{'},S,\lambda }{M}_{x\mathrm{'},y\mathrm{'},S,\lambda }$$

**Fig. 3:**
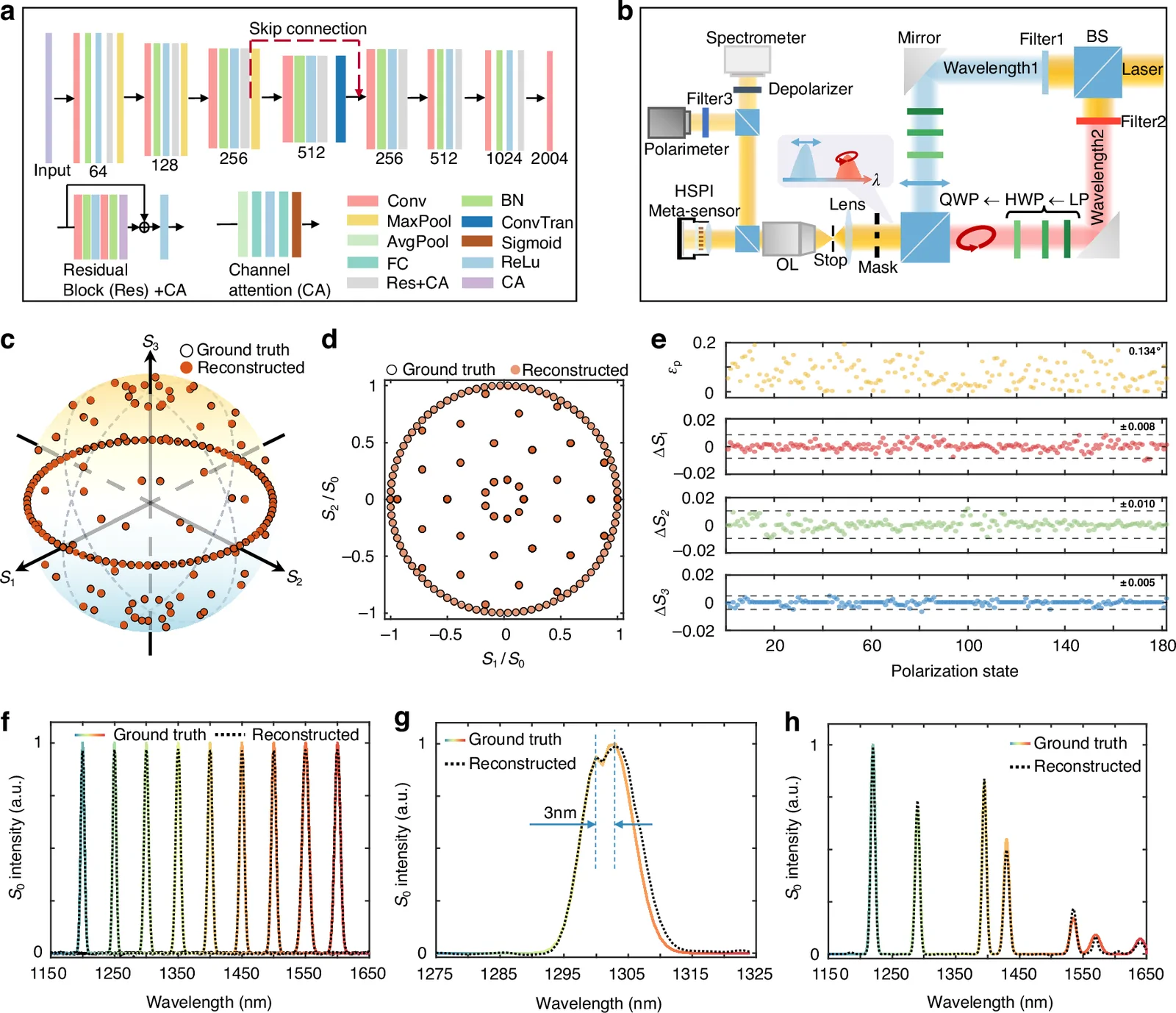
**HSPI-Net architecture and reconstruction performance for polarization and spectra information.** **a** Schematic of the residual attention-based network architecture tailored for hyperspectro-polarimetric imaging (HSPI). Input: a 512 × 512 grayscale image captured by the InGaAs FPA. Hidden layers: a series of residual blocks with spectral-polarization channel attention mechanisms. Output: a 128 × 128 × 2004 tensor (128 × 128 image resolution × 501 spectral channels × 4 Stokes parameters). **b** Schematic of the experimental setup. Polarization state at each wavelength is independently modulated using a linear polarizer, a half-wave plate, and a quarter-wave plate. Spectral state controlled by the input laser and filter1 and filter2, produces light with coupled polarization and wavelength degrees of freedom. **c** Randomly selected reconstructed polarization states (orange dots) and the ground truth (blue circles) distributed over the full Poincaré sphere at a representative wavelength of 1300 nm. **d** Projection of the reconstructed polarization states (orange dots) and the ground truth (black circles) onto the *S*_1_-*S*_2_ plane. **e** Reconstructed errors ${\varepsilon }_{p}$ and the differences between reconstructed and ground-truth Stokes parameters (*S*_1_, *S*_2_, *S*_3_) across 182 polarization states. Standard deviation *σ* is labeled in each subpanel, with dashed lines indicating ±3*σ* confidence intervals. **f** Reconstructed spectra (dashed lines) and the ground truth (solid lines) for nine narrowband spectra within the 1200–1600 nm range at 50 nm intervals, under an arbitrary elliptical polarization state. **g** Reconstructed spectra (dashed lines) and the ground truth (solid lines) of two closely spaced peaks separated by only 3 nm. **h** Reconstructed spectra (dashed lines) and the ground truth (-solid lines) of a complex multi-peak broadband spectrum spanning 1150–1650 nm

${f}_{x,y,x\mathrm{'},y\mathrm{'},S,\lambda }$, which is a sixth-order tensor, represents the spectral-polarimetric encoding governed by the metasurface design. ${M}_{x\mathrm{'},y\mathrm{'},S,\lambda }$, which is a fourth-order tensor, represents the multidimensional optical information of the target. (*x*, *y*) denotes the position on the FPA; (*x’*, *y’*) the position on the object; *S* the Stokes vector; and *λ* the wavelength. To reconstruct ${M}_{x\mathrm{'},y\mathrm{'},S,\lambda }$ from ${I}_{x,y}$, we implement a residual attention-based network to construct a hyperspectral-polarization imaging network (HSPI-net) (Fig. 3a). As shown in Fig. 3a, the network comprises (1) the input layer consisting of a 512 × 512 grayscale image captured by the InGaAs FPA. (2) The hidden layers consist of a series of residual attention-based blocks. The residual structure alleviates the gradient vanishing problem and facilitates stable training of deep networks, while the attention module adaptively emphasizes the most informative spectral-polarization features and suppresses redundant or noisy information, thereby improving feature representation. (3) the output is a three-dimensional tensor of size 128 × 128 × 2004 (128 × 128 image resolution × 501 spectral channels × 4 Stokes parameters, i.e., four-component Stokes vector (*S*_0_, *S*_1_, *S*_2_, *S*_3_) at each wavelength (*λ*_1_, *λ*_2_, …*λ*_n_) spanning the 1150–1650 nm range). Here, the 128 × 128 image resolution is sevenfold times higher than the best-reported value of previous HSPI devices (see SI Note 1 for details).

To train the network, we constructed a large experimental dataset comprising grayscale images captured by the metasurface-empowered InGaAs FPA, along with the corresponding ground-truth HSPI values. The dataset was acquired using a homemade characterization system (Fig. 3b). A supercontinuum laser serves as the broadband light source. An acousto-optic tunable filter (AOTF) was employed to generate a single-peak or multi-peak spectrum across the 1150–1650 nm range. The AOTF enables simultaneous generation of up to 8 narrowband wavelength channels. In addition, several single-mode lasers were also used to expand the spectral diversity of the dataset. The polarization state of the incident beam is precisely controlled using a combination of a linear polarizer, a half-wave plate, and a quarter-wave plate. By rotating these optical elements to specific angles, arbitrary polarization states uniformly distributed over the full Poincaré sphere can be generated. The polarizer and wave plates are mounted on piezoelectric rotation stages to enable programmable control. Two sets of polarization control modules are placed before the beam splitter to independently modulate the polarization states of different optical paths (black brackets in Fig. 3b). This configuration allows the creation of polarization-wavelength intricately coupled light, where different polarization states are assigned to different wavelengths. The light beam is then patterned by a mask, focused by a lens, and then collimated by an objective lens to form the image on the metasurface-empowered InGaAs FPA. Ground-truth values are measured by an infrared polarimeter and a spectrometer (see “Methods” for detailed specifications and calibration procedures). All components (including the InGaAs FPA, piezoelectric rotation stages, and AOTF) are interfaced with a computer and programmed for fully automated operation. This integrated setup enables high-throughput data acquisition and facilitates the collection of a diverse, well-characterized labeled dataset. As a result, the deep learning model can be trained effectively, ensuring accurate and robust reconstruction of HSPI information across varying spatial, spectral, and polarization configurations.

We first demonstrate the reconstruction of polarization states at a narrowband wavelength of 1300 nm. A total of 182 test polarization states (selected from a dataset of 8281 samples, with the remaining 8099 used for training) are reconstructed, and compared with the ground truth on the Poincaré sphere (Fig. 3c). The reconstructed results (orange dots) are highly consistent with the ground truth (black circles), with the polarization states broadly covering the Poincaré sphere. For a better visualization, the Stokes vectors are projected onto the *S*_1_-*S*_2_ plane (Fig. 3d). To quantitatively evaluate the reconstruction performance, we defined the polarization reconstruction error as ${\varepsilon }_{p}=\arccos (\mathop{\sum }\nolimits_{i=1}^{3}({S}_{i}\cdot {{S}_{i}}^{{\prime} })/({S}_{0}\cdot {{S}_{0}}^{{\prime} }))$, where ${S}_{i}$ and ${{S}_{i}}^{{\prime} }$ denote the ground-truth and reconstructed Stokes parameters (*i* = 1, 2, 3), respectively. This metric corresponds to the angle deviation between the ground truth and the reconstructed polarization states on the Poincaré sphere. Our system achieves an average polarization error ${\varepsilon }_{P}$ as low as 0.134°for 182 test cases (Fig. 3e). Additionally, we present the differences between reconstructed and ground-truth Stokes parameters (*S*_1_, *S*_2_, *S*_3_) across all 182 test cases. The deviations fall within ±3*σ* intervals: *S*_1_ = ± 0.008, *S*_2_ = ± 0.010, and *S*_3_ = ± 0.005, as indicated by the black dashed lines in Fig. 3e (*σ* is the standard deviation). This represents the highest polarization reconstruction accuracies reported to date for multidimensional optical sensing (see SI Note 1).

Next, we demonstrate the reconstruction of single-peak spectra at a fixed polarization state. Nine test spectra, each centered at 50 nm intervals, are selected from a total of 5001 single-peak spectra spanning the 1150–1650 nm range; the remaining 4992 spectra are used for training. The reconstruction results for the nine test cases are shown in Fig. 3f. The reconstructed spectra (dashed lines) show good agreement with the ground truth (solid lines). In our system, spectral information is discretized into 501 spectral channels (*λ*_1_, *λ*_2_, … *λ*_501_). The reconstruction error for a spectrum is calculated as the RMSE between the reconstructed and ground-truth spectral values across all channels: ${\varepsilon }_{\lambda }=\sqrt{\mathop{\sum }\nolimits_{i=1}^{501}{({I}_{i}-{{I}_{i}}^{\mathrm{'}})}^{2}/501}$, where ${I}_{i}$ and ${{I}_{i}}^{\mathrm{'}}$ denote the ground-truth and reconstructed spectral information at the *i*th wavelength channel, respectively. For all nine test cases, the spectral reconstruction error is as low as 0.43%.

To evaluate spectral resolution, we generate a series of double-peak spectra. These spectra are formed by combining two light sources: a single-mode laser at 1300 nm and a supercontinuum laser modulated by an AOTF. The polarization states of both sources are controlled to be identical. In addition to the fixed peak of 1300 nm, the supercontinuum-AOTF path provides a tunable narrowband spectral component, enabling dynamic control over the second peak. From a total of 2498 double-peak spectra spanning the 1250–1350 nm range, 50 distinct test cases are selected for spectral resolution evaluation, while the remaining 2448 are used for training. The reconstruction results demonstrate that the system can clearly resolve two spectral peaks with a minimal separation of just 3 nm, corresponding to the highest resolving power (*λ*/Δ*λ* = 433), surpassing the best-reported value of all HSP devices (see SI Note 1). As shown in Fig. 3g, the reconstructed spectra (dashed lines) closely match the ground truth (solid lines), verifying the system’s high spectral resolution.

Furthermore, our system demonstrates accurate reconstruction of more complex spectra containing multiple peaks with varying intensities. These spectra are generated using the 8 simultaneous wavelength channels of the AOTF modulating a supercontinuum laser. Within the spectral range of 1150–1650 nm, a total of 22,815 distinct multi-peak spectra were collected. Among them, 2285 spectra were randomly selected for testing, while the remaining 20,530 were used for training the model. As shown in Fig. 3h, the reconstructed spectra (dashed lines) exhibit excellent agreement with the ground truth measurements (solid lines). Across all 2285 test cases, the spectral reconstruction error remains as low as 2.32%, demonstrating its robustness and accuracy (see additional results in SI Notes 11 and 12).

**Fig. 4:**
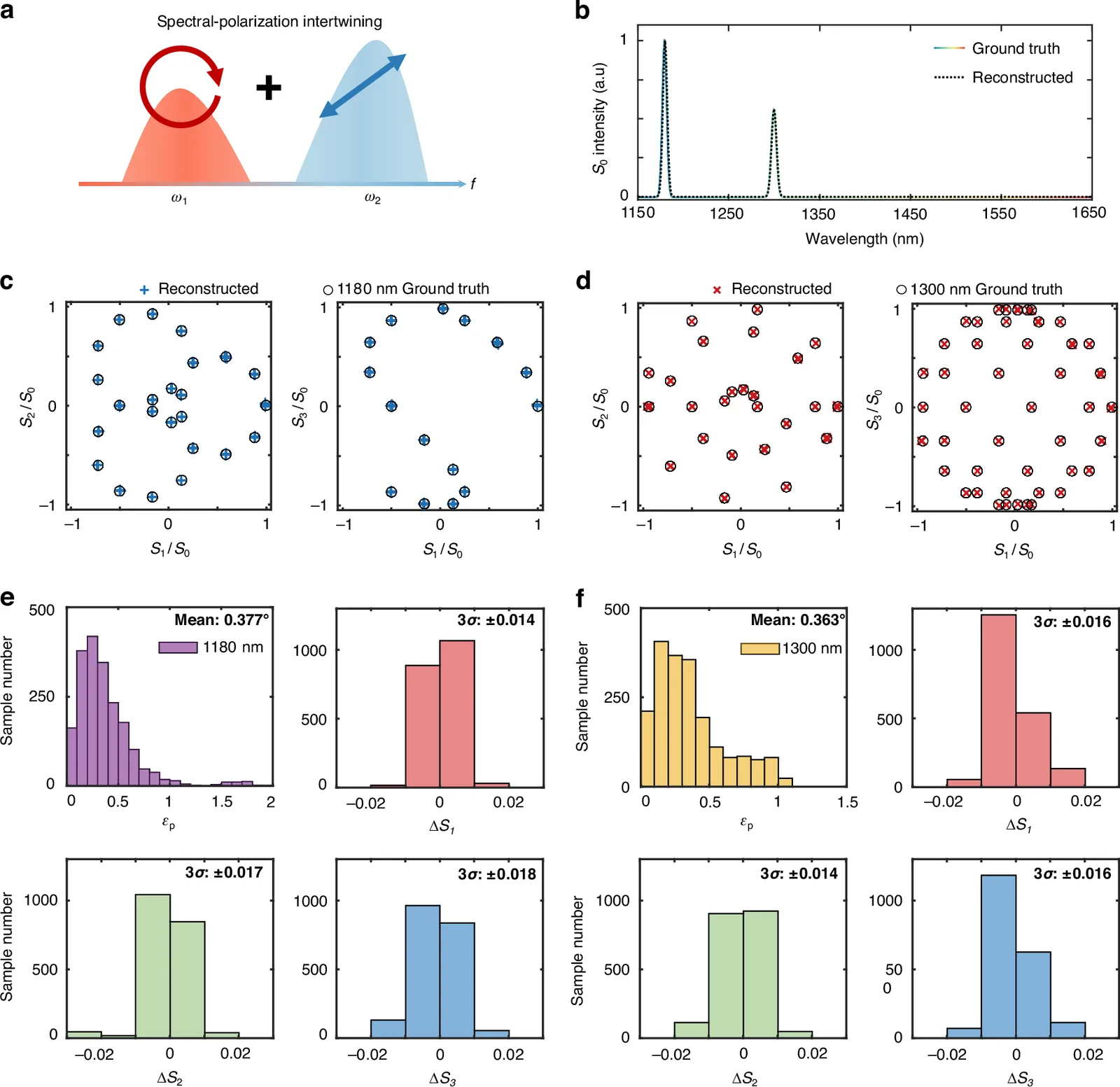
**Sensing of complex polarization states at dual wavelengths.** **a** Illustration of two spectral components carrying distinct polarization states. **b** Reconstructed spectra (dashed lines) and the ground truth (solid lines) for double-peak spectra with different polarization states. **c**, **d** Projection of the reconstructed polarization states (blue or orange dots for 1180 and 1300 nm) and the ground truth (black circles) onto the *S*_1_-*S*_2_ and *S*_1_-*S*_3_ planes. A total of 2000 complex polarization states are presented (40 states at 1180 nm and 50 states at 1300 nm). Reconstructed errors ${\varepsilon }_{p}$ and the differences between reconstructed and ground-truth Stokes parameters (*S*_1_, *S*_2_, *S*_3_) for 2000 polarization states at **e** 1180 nm and **f** 1300 nm. Standard deviation σ is labeled in each subpanel, with dashed lines indicating *±*3*σ* confidence intervals

Although the previous sections have demonstrated accurate reconstruction of either polarization states at fixed wavelengths or spectral information under fixed polarization conditions, real-world optical signals often exhibit more complex behavior, such as spectral-polarization intertwining, where the polarization state varies with wavelength. As schematically illustrated in Fig. 4a, two optical components at different frequencies carrying right-hand circular polarization and 45° linear polarization, respectively, are superimposed. To validate the system’s capability in reconstructing such multidimensional optical information, we present the reconstruction of double-peak spectra, where each spectral peak carries a distinct polarization state. The experimental setup remains the same (Fig. 3b). Two independent polarization control modules are placed before the beam splitter, allowing separate modulation of the polarization states for each optical path. In this experiment, the wavelengths of incident light are 1180 nm and 1300 nm, respectively. Due to the simultaneous variation in both spectral and polarization dimensions, commercial polarimeters or spectrometers alone cannot resolve this information simultaneously. In contrast, our metasurface-empowered InGaAs FPA successfully reconstructs not only the double-peak spectra profiles (Fig. 4b), but also the distinct polarization states corresponding to each peak (Fig. 4c, d). A total of 270,323 wavelength-dependent polarization state samples were collected; 2000 for testing and the remaining for training. As shown in Fig. 4b, the reconstructed spectra (dashed lines) closely match the ground truth (solid lines).

Moreover, the reconstructed polarization states at 1180 and 1300 nm (plotted as blue and orange dots, respectively) show good agreement with the ground truth (black circles) (Fig. 4c, d, the Stokes vector projected onto the *S*_1_-*S*_2_ and *S*_1_-*S*_3_ planes). Quantitatively, the average polarization error ${\varepsilon }_{p}$ at 1180 nm is 0.377°, as shown in Fig. 4e. The differences between the reconstructed and the ground-truth Stokes parameters (*S*_1_, *S*_2_, *S*_3_) across 2000 test samples are within ±3*σ* intervals: *S*_1_ = ± 0.014, *S*_2_ = ± 0.017, and *S*_3_ = ± 0.018, respectively. Similarly, as shown in Fig. 4f for 1300 nm, the average polarization error ${\varepsilon }_{p}$ is 0.363°, with Stokes parameter deviations of *S*_1_ = ±0.016, *S*_2_ = ±0.014, and *S*_3_ = ±0.016.

### Real-time hyperspectral-polarimetric imaging demonstration

Lastly, we demonstrate the real-time capability of our system for HSPI. Beyond reconstructing the hyperspectral-polarimetric information of a simple light spot, our system also demonstrates imaging capability for more complex scenes. By placing a patterned mask (e.g., the shape of the alphabet “8,” “UC,” and “AS”) before the lens, the grayscale image captured by the InGaAs FPA exhibits structured spatial intensity instead of a uniform spot. The raw measurement is a wavelength-dependent polarization encoded intensity pattern, from which our HSPI-net reconstructs a full 4D HSPI data cube containing spatial, spectral, and polarimetric information.

**Fig. 5:**
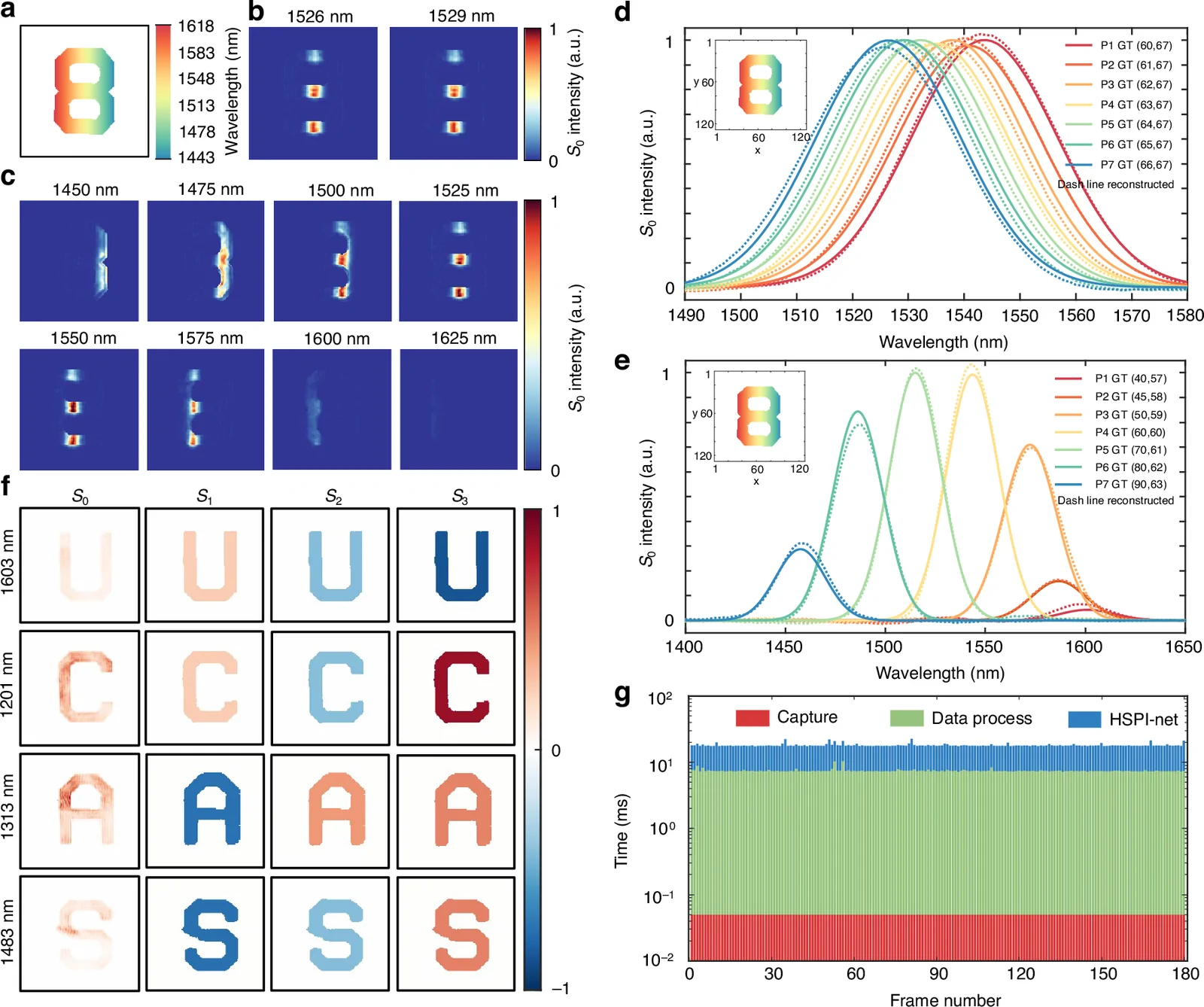
**Real-time hyperspectral-polarimetric imaging demonstration.** **a** Center-wavelength distribution of the HSPI sample. **b** Reconstructed spectral image slices sampled at 3 nm intervals around the peak region. **c** Reconstructed spectral image slices at representative wavelengths, sampled at 25 nm intervals. **d** Pixel-wise reconstructed spectral profiles for two neighboring pixels (separated by one pixel step, marked in the inset), exhibiting a 3 nm shift in their center wavelengths, reconstructed spectra (dashed lines), and the ground truth (solid lines). **e** Pixel-wise reconstructed spectral profiles for a larger spatial separation (marked in the inset), reconstructed spectra (dashed lines), and the ground truth (solid lines). **f** Reconstructed Stokes images at representative center wavelengths for different targets, demonstrating simultaneous recovery of spatial and polarimetric information. **g** Timing breakdown over video frames, including acquisition (capture), preprocessing (data processing), and HSPI-net inference (HSPI-net) for 180 test instances

In our system, the effective operating bandwidth is 500 nm, while the equivalent spectral resolution—defined as the distinguishable center-wavelength step or channel spacing—is about 3 nm. This corresponds to 167 channels across the operating band. In order to demonstrate the capability of our method to deal with complex scenarios, a linear variable filter (LVF) is used to introduce spatially varying spectra along one sensor direction. We collected 78 LVF-based hyperspectral samples: 76 were used to train the HSPI-net, and the remaining 2 samples were used for inference. Figure 5a shows the center-wavelength distribution of a representative test sample over 1443–1618 nm, indicating dense and quasi-continuous wavelength sampling rather than a small number of discrete spectral bands. As illustrated by the 3 nm-step image slices in Fig. 5b, the system reliably reconstructs narrowband 2D images at fine spectral increments. This performance enables the formation of a high-density, quasi-continuous spectral cube, fulfilling the fundamental criteria for multi-channel hyperspectral imaging. In addition, Fig. 5c shows 2D spectral image slices sampled with a 25 nm step, characterizing the transition of the spatial features across a wide band. More importantly, to verify that the output is not only visually reasonable at the slice level but also spectrally consistent and discriminative at the pixel level, we further perform pixel-wise spectral-curve validation. Figure 5d compares the reconstructed spectral profiles of 7 adjacent pixels along the *x*-direction with the ground truth (measured by a microscope equipped spectrometer). Notably, the reconstructed spectra exhibit a center-wavelength spacing of 3 nm between adjacent pixels. Figure 5e presents the reconstructed spectra and the ground truth of 7 pixels that are far apart. Similar to the case of adjacent pixels (Fig. 5d), the reconstructed spectra closely match the ground truth, demonstrating high-fidelity recovery of spatially diverse spectral signatures.

To quantitatively assess reconstruction performance, we compute the RMSE between the reconstructed HSPI data cube and the ground truth:

$${\varepsilon }_{HSPI}=\sqrt{\mathop{\sum }\limits_{{\rm{i}},j=1}^{128}\left(\mathop{\sum }\limits_{u=1}^{501}\left(\mathop{\sum }\limits_{v=0}^{3}{({x}_{i,j,u,v}-{x{\prime} }_{i,j,u,v})}^{2}\right)\right)/(128\times 128\times 501\times 4)}$$

where *x* and *x*’ represent reconstructed and ground-truth multidimensional HSPI data, respectively. Across the test data, we obtain an average RMSE is 1.731%. indicating high numerical consistency over the full 4D cube (spatial-spectral-polarimetric).

Beyond spectral reconstruction, the system simultaneously recovers polarization information (the polarization information of Fig. 5a, see the SI Video). To further validate the polarization reconstruction capability, we collected a total of 199528 wavelength-dependent polarization-state samples, among which 4802 were used for testing and the remaining samples for training. Figure 5f further presents polarization-resolved Stokes image slices (*S*_0_, *S*_1_, *S*_2_, *S*_3_) of different targets (e.g., “UC” and “AS”) at representative central wavelengths. The reconstructed spatial structures and Stokes components closely match the ground-truth measurements with an average RMSE of only 0.177%, demonstrating high-fidelity Stokes parameters reconstruction (Fig. 5f, top: 1603 nm for “U,” 1201 nm for “C,” 1313 nm for “A,” and 1483 nm for “S”).

Finally, we summarize the computational cost of the HSPI reconstruction pipeline over 180 frames (Fig. 5g). The results show that camera acquisition plus data preprocessing and HSPI-net inference each account for approximately 50% of the total runtime. Under our current computing setup (see “Methods”), the average per HSPI instance time is 18 ms, enabling a real-time throughput of ~55 frames of HSPI per second.

## Discussion

The HSPI-net successfully reconstructs high-dimensional data from sparse measurements by learning the deterministic physical mapping of the metasurface rather than memorizing training samples (see SI Note 13 for details). Quantitative evaluation using the nearest-neighbor similarity score confirms that the network maintains high fidelity even for unseen data with low similarity to the training set. This proves that the system effectively leverages learned physical priors to generalize across diverse spectral-polarimetric distributions, ensuring robust performance beyond the specific constraints of the training distribution (see SI Note 14 for details).

Building on this strong generalization capability, we further discuss the scalability of HSPI-net in terms of spatial resolution and spectral coverage. Owing to its fully convolutional, translation-invariant backbone, higher-resolution inputs can be processed via patch-based/sliding-window inference using the same pre-trained weights, with computation scaling approximately linearly with pixel count. Moreover, the current output size is a task-specific setting rather than an architectural constraint: bandwidth shifts can be accommodated with the same network and brief fine-tuning, while changes in spectral sampling density mainly require reconfiguring the final outputs, reusing the backbone via weight transfer.

In addition, our approach has a good tolerance toward fabrication imperfections. Although fabrication imperfections inevitably introduce systematic perturbations, such as increased 0th-order diffraction and inter-channel crosstalk, the end-to-end HSPI-net learns the effective system response directly from training data. As a result, these non-idealities are inherently incorporated into the learned forward model, enabling the network to compensate for consistent and learnable error patterns within realistic manufacturing tolerances. Consequently, the final reconstruction remains robust in the presence of realistic fabrication deviations, highlighting the synergy between physical encoding and deep-learning decoding (see SI Note 15 for details).

Finally, to validate feasibility under resource-constrained conditions, we executed HSPI-net directly on a mobile/edge-class embedded processor without using any external desktop GPU. The HSPI-net successfully accomplishes an on-device end-to-end forward pass and produces the full output tensor. This effort confirms that the HSPI-net is fundamentally deployable on resource-limited hardware, with clear headroom for further acceleration toward practical real-time operation in compact imaging systems (see SI Note 16 for details).

We have demonstrated a compact, deep-learning-empowered HSPI device capable of simultaneous, instantaneous, and high-accuracy characterization of multidimensional light information. Our approach achieves unprecedented spectral and full-Stokes polarization reconstruction accuracy across the broad range of 1150–1650 nm. The key performance metrics—including spectral and image resolution, as well as spectral and polarization reconstruction accuracy—are superior to the best-reported specifications of hyperspectro-polarimetric cameras, with enhancements ranging from several-fold to one order of magnitude. The acquisition, processing, and inference time only takes 18 ms, allowing high-dimensional hyperspectral-polarimetric imaging at 55 frames per second in real time. This work establishes a versatile and scalable platform for high-dimensional optical sensing, offering new opportunities for applications in real-time multidimensional light-field analysis.

## Methods

### Numerical simulation

The optical responses of nanopillars were simulated with a single unit and applying periodic boundary conditions, using the finite-difference time-domain method (FDTD Solutions package, Lumerical). The light source was a plane wave with normal incidence from the air. The boundaries above and below the nanopillars were perfectly matched layers. From bottom to top, the simulated structure consists of a silicon layer (1000 nm thickness) and a sapphire substrate.

### Sample fabrication

The fabrication of the metasurface was done via ShanghaiTech Material and Device Lab (SMDL). The fabrication process is schematically shown in Supplementary Fig. S4. The metasurface array was first patterned on a 1000-nm-thick monocrystalline silicon film on a sapphire substrate by the electron beam lithography using a negative tone resist, hydrogen silsesquioxane (HSQ). The next step involved transferring the pattern onto the silicon layer using HSQ as the mask via the reactive ion etching process. Finally, the HSQ resist was removed by buffered oxide etchant.

### Experimental setup

For experimental characterization, the input light is generated by coupling a supercontinuum light source (YSL SC-Por) with an AOTF. The AOTF produces narrowband Gaussian-shaped light, and by simultaneously activating multiple channels, it can generate broadband multi-peak spectra. A linear polarizer is used to initialize the polarization state, while a half-wave plate and a quarter-wave plate are employed to generate arbitrarily polarized light covering the full-Stokes parameters (Fig. 3b). An objective lens (Mitutoyo Plan Apo Infinity Corrected Long Working Distance Objective, 20×) is used to introduce and collect light with various incident and azimuthal angles. After passing through a focusing lens (Thorlabs AL2520, focal length 20 mm), the light is captured by an InGaAs FPA (Bobcat 640, 640 × 512 pixels, 15 μm pixel resolution, operating wavelength range 0.7–1.7 μm), resulting in spectro-polarimetric encoded images.

### Model training details

As illustrated in the schematic of the HSPI network model in Fig. 3a, the input layer receives mapped grayscale image matrices measured under arbitrary combinations of polarization and spectral states. The output layer provides a 128 × 128 × 2004 tensor (predicted values), representing the spatial resolution 128 × 128 and four Stokes parameters (S0, S1, S2, S3) at 501 discrete wavelengths. When needed, a tanh activation function can be introduced at the output layer to constrain the predictions within the range of −1 to 1, to match the normalized polarization parameter values of S0, S1, S2, and S3. For network training, the Adam optimizer is utilized with an initial learning rate of 1e-3, and a cosine annealing learning rate scheduler (CosineAnnealingLR) is implemented in PyTorch. The network adopts the SmoothL1Loss as its loss function. The acquired images are cropped to a resolution of 512 × 512 and subsequently undergo denoising and normalization. And the spectra collected by the spectrometer are filtered using Gaussian convolution to suppress sidelobes and noise. All network training and inference were conducted on a workstation equipped with an Intel Xeon 8473C CPU and an NVIDIA RTX 5880 Ada GPU with 48GB VRAM.

### High-dimensional data inference

To demonstrate the potential for inference-stage optimization, we implemented an efficiency-oriented inference pipeline for HSPI-net while strictly preserving the input/output tensor resolution and channel ordering. Inference is executed on the GPU in evaluation mode under inference mode, and we enable cuDNN’s fixed-shape optimization to improve convolution kernel selection for constant input sizes. For BN-containing blocks, we perform Conv-BatchNorm fusion (merging adjacent Conv2d-BatchNorm2d pairs under eval, which is functionally equivalent at inference) to reduce operator overhead. In addition, we employ automatic mixed precision via FP16 autocasting on CUDA to alleviate compute and memory-bandwidth pressure during convolutions and tensor operations; this does not alter the output dimensions or channel permutation.

Based on the same hardware platform, the original (unoptimized) HSPI-net runs at an average throughput of ~4 fps, whereas the optimized inference pipeline improves the throughput to 55 fps.

## Supplementary information

Supplement Material

HSPI Video of Fig 5 LVF

HSPI Video of Fig 5 UCAS

## Data Availability

The data that support the plots in this paper are available from the corresponding authors. Source data are provided with this paper. The demo code of this work is available from the public repository at https://github.com/faiminlaw/HSPI.
