# Supplementary material for: High-accuracy hyperspectro-polarimetric real-time imaging via a deep learning empowered infrared meta-sensor
Source: Light Sci Appl. 2026 Aug 20;15:352. doi: 10.1038/s41377-026-02407-1 (PMC13493852; doi:10.1038/s41377-026-02407-1)
Supplement: Supplementary file 1 [file 41377_2026_2407_MOESM1_ESM.pdf]

Supplementary Information for "High-accuracy hyperspectro-polarimetric real-time imaging via a deep learning empowered infrared meta-sensor"

Huiming Luo[‡1,2,3],Jie Deng[‡1], Jing Zhou[*1,3,4], Tianyuan Cui[1,5] , Yuejie zhang[1,3], Junwei Huang[1,3], Liwen Fu[1,3], Yonghao Bu[1] , Ruowen Wang[1,3], Mengdie Shi[1,3], Xiaofei He[1,3], Xueling Guo[1,3], Jun Ning[1,3], Xiaoshuang Chen[*1,2,3,6] & Wei Lu[*1,2,3,5]

1. State Key Laboratory of Infrared Physics, Shanghai Institute of Technical Physics, Chinese Academy of Sciences, Shanghai 200083, China.
2. School of Physics and Optoelectronic Engineering, Hangzhou Institute for Advanced Study, University of Chinese Academy of Sciences, Hangzhou 310024, China.
3. University of Chinese Academy of Sciences, Beijing 101408, China.
4. Shanghai Key Laboratory of Optical Coatings and Spectral Modulation, Shanghai Institute of Technical Physics, Chinese Academy of Sciences, Shanghai 200083, China.
5. School of Physical Science and Technology, ShanghaiTech University, Shanghai 201210, China
6. Suzhou Laboratory, Suzhou 215000, Jiangsu, China

‡ These authors contributed equally: Huiming Luo, Jie Deng.

* Corresponding authors: Jing Zhou, Xiaoshuang Chen, Wei Lu.

Corresponding author E-mail: jzhou@mail.sitp.ac.cn; xschen@mail.sitp.ac.cn; luwei@mail.sitp.ac.cn

Note 1 Comparisons of spectral-polarimetric reconstruction performance..............................2
Note 2 Optical response of nanopillars and metasurface design..4
Note 3 Derivation of the metasurface structure design 5
Note 4 Far-field simulation of the spectro-polarimetric encoding 8
Note 5 Fabrication processes 10
Note 6 Experiment setup...10
Note 7 Central light leakage fabrication imperfections.. 11
Note 8 System field of view (FOV) 14
Note 9 Imaging spatial resolution 18
Note 10 Metasurface efficiency 19
Note 11 Implement of the deep learning 20
Note 12 Extended reconstruction results..22
Note 13 Sparse sampling dataset...25
Note 14 Generalization capability...27
Note 15 HSPI-net for compensating for device errors 29
Note 16 Cross-platform deployment and on-device validation on an embedded system........31

Note 1 Comparisons of spectral-polarimetric reconstruction performance

Reference	work Wavelength (μm)	Bandwidth	Imaging capability	Image resolution* (pixels)	Double-Peak Spectral Resolution ($\Delta\lambda$)	Spectral Resolution power ($\lambda/\Delta\lambda$)	Spectral Reconstruction Error	
							L1 norm	RMSE
Ref.37(2022)	1.4-1.5	100 nm	×	–	30 nm	48	7.80 %	–
Ref.38(2023)	0.65-0.70	50 nm	×	–	–	–	–	–
Ref.36(2024)	0.5-0.7	200 nm	×	–	8.7 nm	75	–	1.87 %
Ref.44(2024)	0.4-0.9	500 nm	√	19 × 14	2.7 nm	196	5.85 %	1.87 %
Ref.45(2024)	0.7-1.15	450 nm	√	42 × 50	–	–	–	–
Ref.41(2025)	0.8-1.1	300 nm	×	–	–	71	77.69 %	–
Ref.35(2025)	0.69-0.83	140 nm	×	–	6 nm	126	-	–
This work	1.15-1.65	500 nm	√	128 × 128	3 nm	433	7.17 % (main text fig.3h)	0.41 % (main text fig.3h)

Image resolution *:

Ref.38(2024): The microlens array placed in front of the camera is used for imaging. Each microlens has a size of 0.8 mm, while the Orion OR-455BSI (SONY IMX455, 4096 × 3000) camera features a pixel size of 3.76 μm. This corresponds to a sensor area of 15.4 mm × 11.2 mm. Based on this configuration, the effective image resolution is calculated to be approximately 19 × 14 pixels.

**Reference**	**Polarization State**	**Average** $\left|\frac{\Delta S_i}{S_i}\right| i = 1,2,3$	$\boldsymbol{\varepsilon_p = cos^{-1}(\frac{\sum_{i=1}^{3} S_i S_i'}{norm(S_0) * norm(S_0')})}$
Ref.37(2022)	Elliptically	-	5°
Ref.38(2023)	Full Stokes	-	7°
Ref.36(2024)	Elliptically	-	4°(estimate)
Ref.44(2024)	Full Stokes	5.87 %	1.33°(estimate)
Ref.45(2024)	Full Stokes	-	10°
Ref.41(2025)	Linearly	-	4.76°
Ref.35(2025)	Full Stokes	-	1.69°
This work	Full Stokes	0.62 %	0.14 °

Note 2 Optical response of nanopillars and metasurface design

Full-wave numerical simulations using the finite-difference time-domain (FDTD) method were conducted to analyze the optical response (phase shifts and transmission efficiency) of individual nanopillars. Each unit cell consists of an amorphous silicon (α-Si) nanopillar fabricated on an alumina (Al_2O_3) substrate. Simulations were carried out under two orthogonal polarization state of incident light of wavelength of 1.3 μm. Periodic boundary conditions were applied around the unit cell, which has a fixed period of 560 nm. The nanopillars height was set to 1000 nm, while its length (Du) and width (Dv) were varied from 138 nm to 426 nm in 2 nm increments. Based on the simulation results (Fig. S1), meta-atoms exhibiting high transmission efficiency and strong polarization-dependent phase modulation were selected and compiled into a design library for metasurface implementation. To spatially multiplex incident light with different polarization states into distinct channels, the phase profile of the metasurface is carefully designed, as shown in Fig. S2.

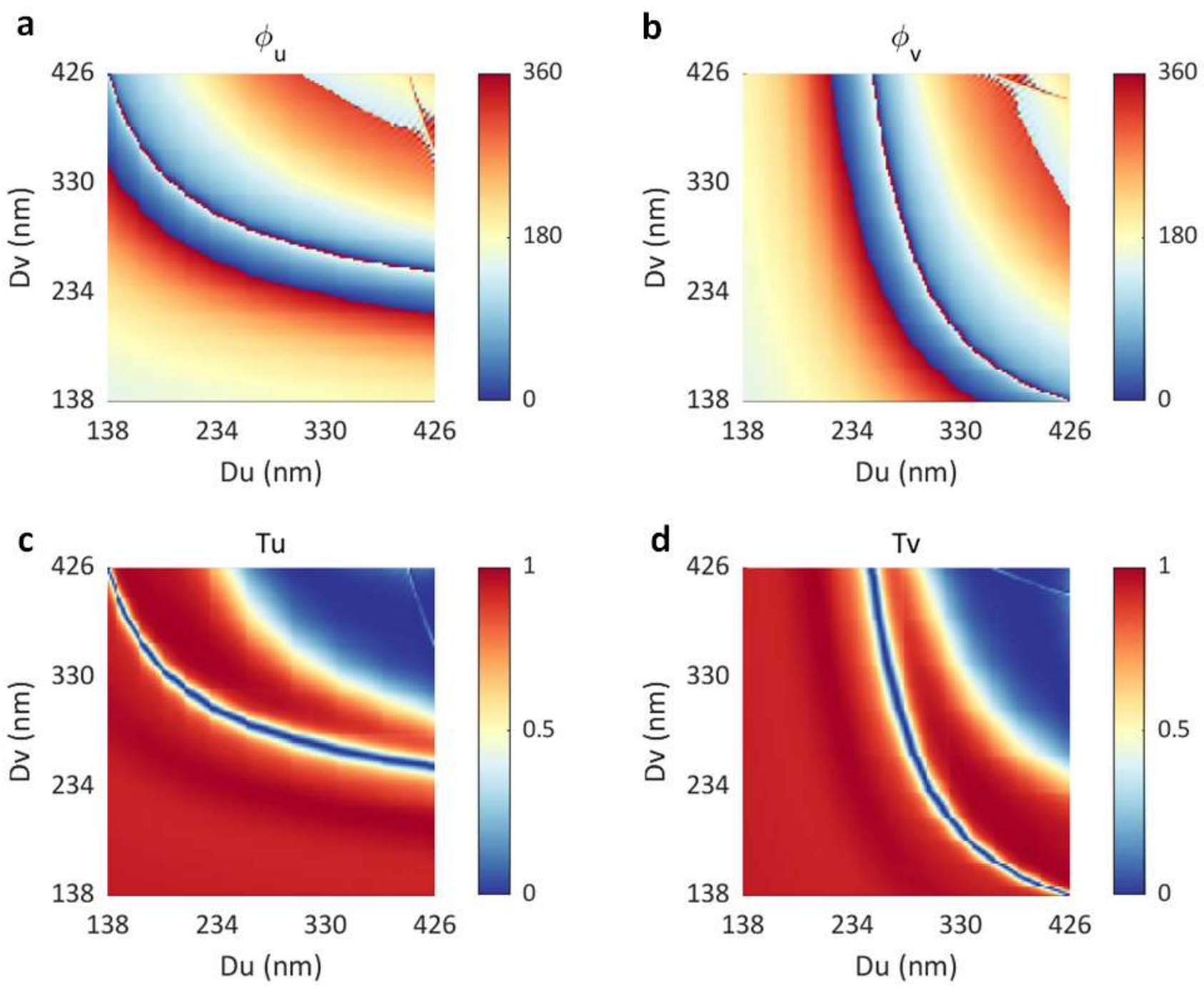

Figure S1 (a-b) Simulated phase shifts (φu and φv) and **(c-d)** transmission efficiency (Tu and Tv) along ordinary and extraordinary axes, respectively, as a function of meta-atom dimensions at a wavelength of 1.3 μm.

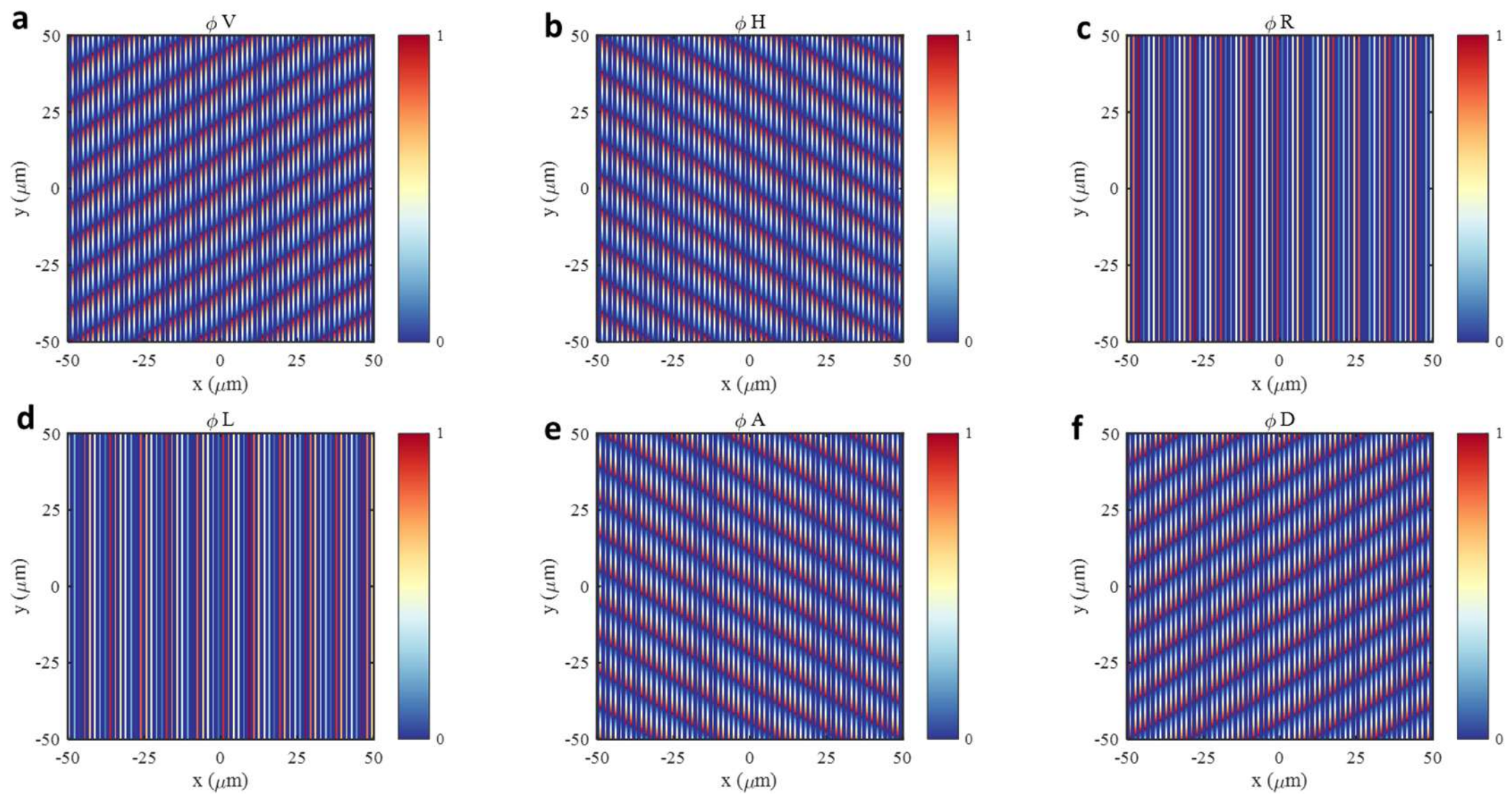

Figure S2 Designed phase profiles for spatially multiplexing incident light under six polarization states: H, V, A, D, L and R.

Note 3 Derivation of the metasurface structure design

The transmission of incident light through a nanofin located at position (x, y) can be described using Jones calculus as:

$$|E_t\rangle = J(x,y)|E_i\rangle \tag{S1}$$

where the incident and transmitted electric fields in the orthogonal coordinate system (x, y) are:

$$|E_i\rangle = \begin{bmatrix} E_{ix} \\ E_{iy} \end{bmatrix}$$

$$|E_t\rangle = \begin{bmatrix} E_{tx} \\ E_{ty} \end{bmatrix} \tag{S2}$$

For a unit cell whose short/long axes are aligned with the along the x- and y-axis, the Jones matrix take the diagonal form:

$$J = \begin{bmatrix} t_u & 0 \\ 0 & t_v \end{bmatrix} \tag{S3}$$

where, t_u、t_v are the complex transmission coefficients along the ordinary and extraordinary axes of the anisotropic meta-atom, respectively.

To enable polarization-selective phase modulation, we assign different phase shifts

$\Phi_u(x,y)$ and $\Phi_v(x,y)$, while keeping the transmission amplitudes equal (Tu = Tv = T). The corresponding Jones matrix becomes:

$$J_0(x,y) = \begin{bmatrix} T_u(x,y)e^{i\Phi_u(x,y)} & 0 \\ 0 & T_v(x,y)e^{i\Phi_v(x,y)} \end{bmatrix} \quad (S4)$$

If the nanopillar is rotated by an angle of θ, the rotated Jones matrix is given by:

$$J(x,y) = R\big(-\theta(x,y)\big) \cdot J_0(x,y) \cdot R(\theta(x,y)) \quad (S5)$$

Where the 2×2 rotation matrix is defined as:

$$R(\theta) = \begin{bmatrix} cos\theta & -sin\theta \\ sin\theta & cos\theta \end{bmatrix} \quad (S6)$$

After algebraic simplification, the general expression for the Jones matrix (JG) of a rotated anisotropic meta-atom becomes:

$$J_G(x,y) = Te^{i\Phi} \begin{bmatrix} cos\frac{\delta}{2} + icos2\theta sin\frac{\delta}{2} & -isin2\theta sin\frac{\delta}{2} \\ -isin2\theta sin\frac{\delta}{2} & cos\frac{\delta}{2} - icos2\theta sin\frac{\delta}{2} \end{bmatrix} \quad (S7)$$

Where, $\Phi = \frac{(\Phi_u+\Phi_v)}{2}$ is the average phase shifts, and $\delta = \Phi_u - \Phi_v$ is the phase retardance between the two orthogonal polarization states.

To analyze and characterize the optical response of the metasurface under different polarization bases, we present the Jones vectors and corresponding matrix expressions for three common pairs of orthogonal polarization states:

(1): horizontal linear polarization (H, 0°) and vertical linear polarization (V, 90°);

(2): anti-diagonal linear polarization (A, 135°) and diagonal linear polarization (D, 45°);

(3): left-handed circular polarization (L) and right-handed circular polarization (R).

For H and V polarization, the incident electric field can be expressed as:

$$\begin{bmatrix} E_H \\ E_V \end{bmatrix} = \begin{bmatrix} 1 & 0 \\ 0 & 1 \end{bmatrix} \begin{bmatrix} E_x \\ E_y \end{bmatrix} \quad (S8)$$

The Jones matrix corresponding to H and V polarizations is given by:

$$J_{HV}(x,y) = \begin{bmatrix} 1 & 0 \\ 0 & 1 \end{bmatrix} J_G \begin{bmatrix} 1 & 0 \\ 0 & 1 \end{bmatrix}^{-1} \quad (S9)$$

After simplification by introducing the average phase $\Phi = \frac{\Phi_u+\Phi_v}{2}$ and the phase retardance $\delta = \Phi_u - \Phi_v$, the Jones matrix becomes:

$$J(x,y) = t \begin{bmatrix} e^{i\Phi_u}\cos^2 2\theta + e^{i\Phi_v}\sin^2 2\theta & \left(e^{i\Phi_u} - e^{i\Phi_v}\right)sin2\theta cos2\theta \\ \left(e^{i\Phi_u} - e^{i\Phi_v}\right)sin2\theta cos2\theta & e^{i\Phi_u}\sin^2 2\theta + e^{i\Phi_v}\cos^2 2\theta \end{bmatrix} \quad (S10)$$

When considering only the propagation phase and setting $\theta = 0$, the Jones matrix simplifies to:

$$J(x,y) = t\begin{bmatrix} e^{i\Phi_u} & 0 \\ 0 & e^{i\Phi_v} \end{bmatrix} \qquad (S11)$$

which means that in this situation, only the propagation phase is considered.

For D and A polarization, the incident electric field can be expressed as:

$$\begin{bmatrix} E_D \\ E_A \end{bmatrix} = \frac{1}{\sqrt{2}}\begin{bmatrix} 1 & 1 \\ 1 & -1 \end{bmatrix}\begin{bmatrix} E_x \\ E_y \end{bmatrix} \qquad (S12)$$

The Jones matrix corresponding to H and V polarizations is given by:

$$J_{DA}(x,y) = \frac{1}{2}\begin{bmatrix} 1 & 1 \\ 1 & -1 \end{bmatrix} J_G \begin{bmatrix} 1 & 1 \\ 1 & -1 \end{bmatrix}^{-1} \qquad (S13)$$

After simplification by introducing the average phase $\Phi = \frac{\Phi_u+\Phi_v}{2}$ and the phase retardance $\delta = \Phi_u - \Phi_v$, the Jones matrix becomes:

$$J(x,y) = t\begin{bmatrix} e^{i\Phi_u}\cos^2(\theta+\frac{\pi}{4}) + e^{i\Phi_v}\sin^2(\theta+\frac{\pi}{4}) & \frac{1}{2}\left(e^{i\Phi_u} - e^{i\Phi_v}\right)sin2\left(\theta+\frac{\pi}{4}\right) \\ \frac{1}{2}\left(e^{i\Phi_u} - e^{i\Phi_v}\right)sin2\left(\theta+\frac{\pi}{4}\right) & e^{i\Phi_u}\sin^2(\theta+\frac{\pi}{4}) + e^{i\Phi_v}\cos^2(\theta+\frac{\pi}{4}) \end{bmatrix} (S14)$$

This matrix can be mathematically represented as the sum of the rotation angle θ and an additional π/4 phase term. This implies that, for the D and A linearly polarized incident light channels, achieving the target phase modulation requires meta-atoms uniformly oriented at θ = π/4. Consequently, a complete phase coverage spanning 0 to 2π must be realized by the meta-atoms with this fixed orientation, enabling full and continuous control over the transmitted phase profile for these specific linear polarization states.

For RCP (R) and LCP (L) polarized light, the incident electric field can be expressed as:

$$\begin{bmatrix} E_R \\ E_L \end{bmatrix} = \frac{1}{\sqrt{2}}\begin{bmatrix} 1 & i \\ 1 & -i \end{bmatrix}\begin{bmatrix} E_x \\ E_y \end{bmatrix} \qquad (S15)$$

$$J_{RL}(x,y) = \frac{1}{2}\begin{bmatrix} 1 & i \\ 1 & -i \end{bmatrix} J_G \begin{bmatrix} 1 & i \\ 1 & -i \end{bmatrix}^{-1} \qquad (S16)$$

After simplification by introducing the average phase $\Phi = \frac{\Phi_u+\Phi_v}{2}$ and the phase retardance $\delta = \Phi_u - \Phi_v$, the Jones matrix becomes:

$$J(x,y) = t\begin{bmatrix} e^{i\Phi_u} + e^{i\Phi_v} & (e^{i\Phi_u} - e^{i\Phi_v})e^{-i2\theta} \\ (e^{i\Phi_u} - e^{i\Phi_v})e^{i2\theta} & e^{i\Phi_u} + e^{i\Phi_v} \end{bmatrix} \qquad (S17)$$

The symmetry of the matrix indicates that the cross-polarized component shares a common propagation phase and a geometric phase governed by ±2θ, which are

determined by the geometry and orientation of the meta-atoms, respectively.

Note 4 Far-field simulation of the spectro-polarimetric encoding

To validate the spectro-polarimetric encoding capability to the metasurface, we perfrome full-wave simulations of the far-field intensity distributions under illumination with various linearly and circularly polarization states over the wavelengths range of 1.15 to 1.65 μm (Figure S3 a and d). For each polarization channel, the integrated intensity within a region defined by a diameter of three times full width at half maximum (FWHM) was extracted for quantitative analysis. Using the definition of Stokes parameters ($S_0 = I_H + I_V,\ S_1 = I_H - I_V,\ S_2 = I_D - I_P,\ S_3 = \ I_{LCP} + I_{RCP}$), we reconstructed the polarization states at six representative wavelengths: 1.15, 1.25, 1.35, 1.45, 1.55, and 1.65 μm. Figure S3 g and h summarize the reconstructed Stokes parameters projected onto the S1-S2, S1-S3, S2-S3 planes, compared to the theoretical values.

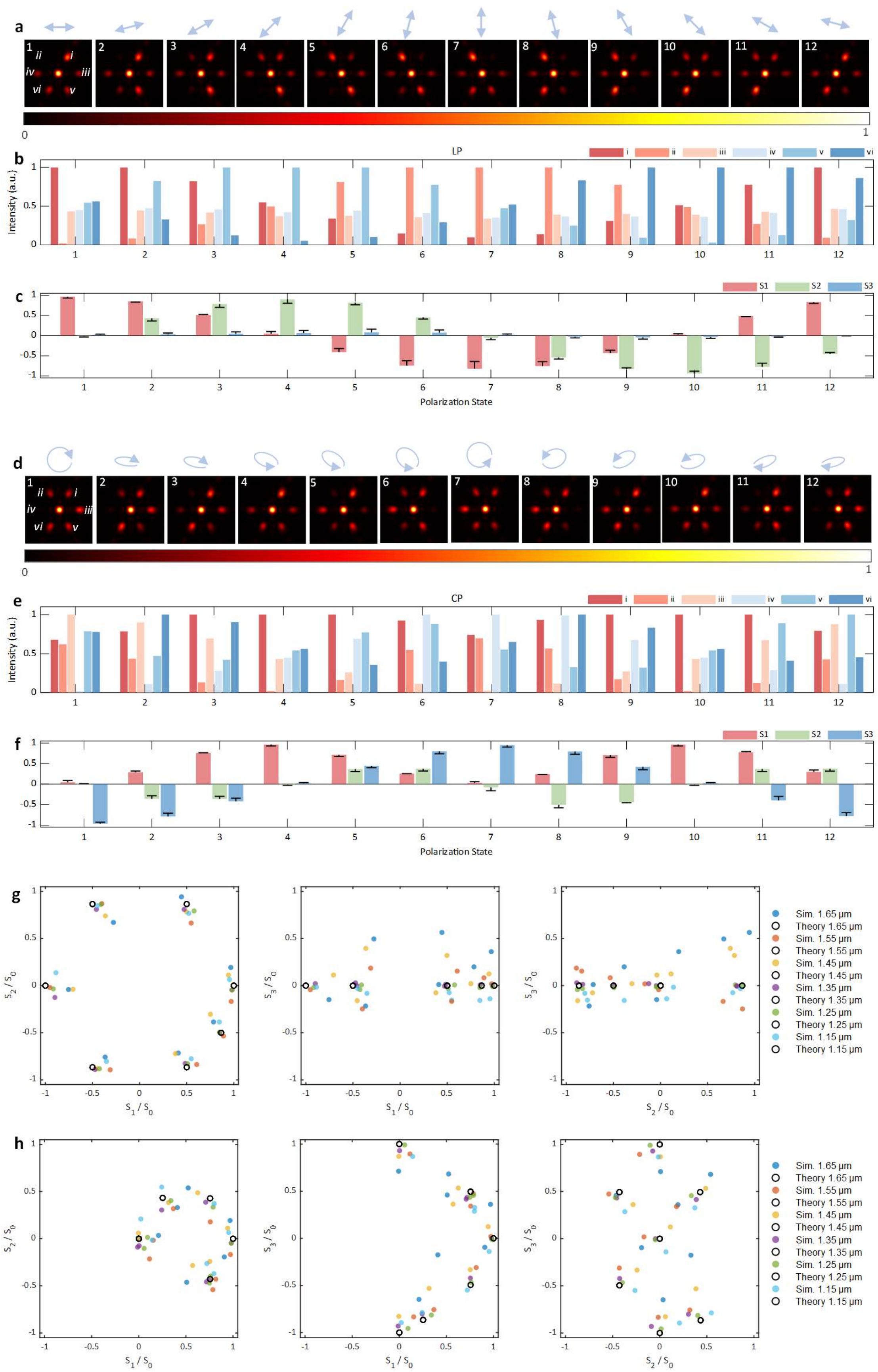

Figure S3. Simulation results for incident light of linearly and circularly polarization states over wavelengths ranging from 1.15 to 1.65 μm. (a) Far-field intensity

distribution under illumination of linearly polarization states. (b) Normalized optical intensities across six polarization channels for the polarization state in (a). (c) Reconstructed Stokes parameters (S_1, S_2, S_3) based on definition, compared with theoretical ground truth (black line) for the polarization states in (a). (d) Far-field intensity distributions under illumination of polarization states with varying light helicities. (e) Normalized optical intensities across six polarization channels for polarization states in (d). (f) Reconstructed Stokes parameters (S_1, S_2, S_3) based on definition, compared with theoretical ground truth (black line) for polarization states in (d). (g) and (h) Reconstructed Stokes parameters (S_1, S_2, S_3) projected onto S_1-S_2, S_1-S_3, S_2-S_3 planes for incident wavelengths of 1.15, 1.25, 1.35, 1.45, 1.55 and 1.65 um.

Note 5 Fabrication processes

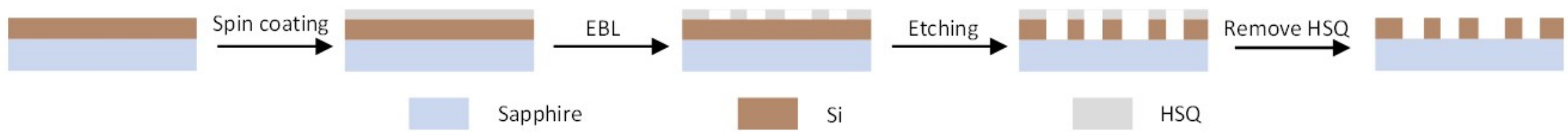

Figure S4. Schematic of the metasurface fabrication process. EBL: electron beam lithography; HSQ: hydrogen silsesquioxane, a negative-tone photoresist.

Note 6 Experiment setup

The designed metasurface is integrated a near-infrared InGaAs focal plane array (FPA) and coupled with an aspheric lens. The polarization state of the incident beam is precisely controlled using a combination of a linear polarizer, a half-wave plate, and a quarter-wave plate. The beam then passes through a custom-designed mask and is focused before reaching the objective lens (OL), where an aperture stop is used to suppress stray light. A beamsplitter (BS) subsequently divides the light into two paths: one directed toward the metasurface-integrated imaging system, and the other routed to a commercial spectrometer or polarimeter to obtain ground-truth spectral or polarization measurements.

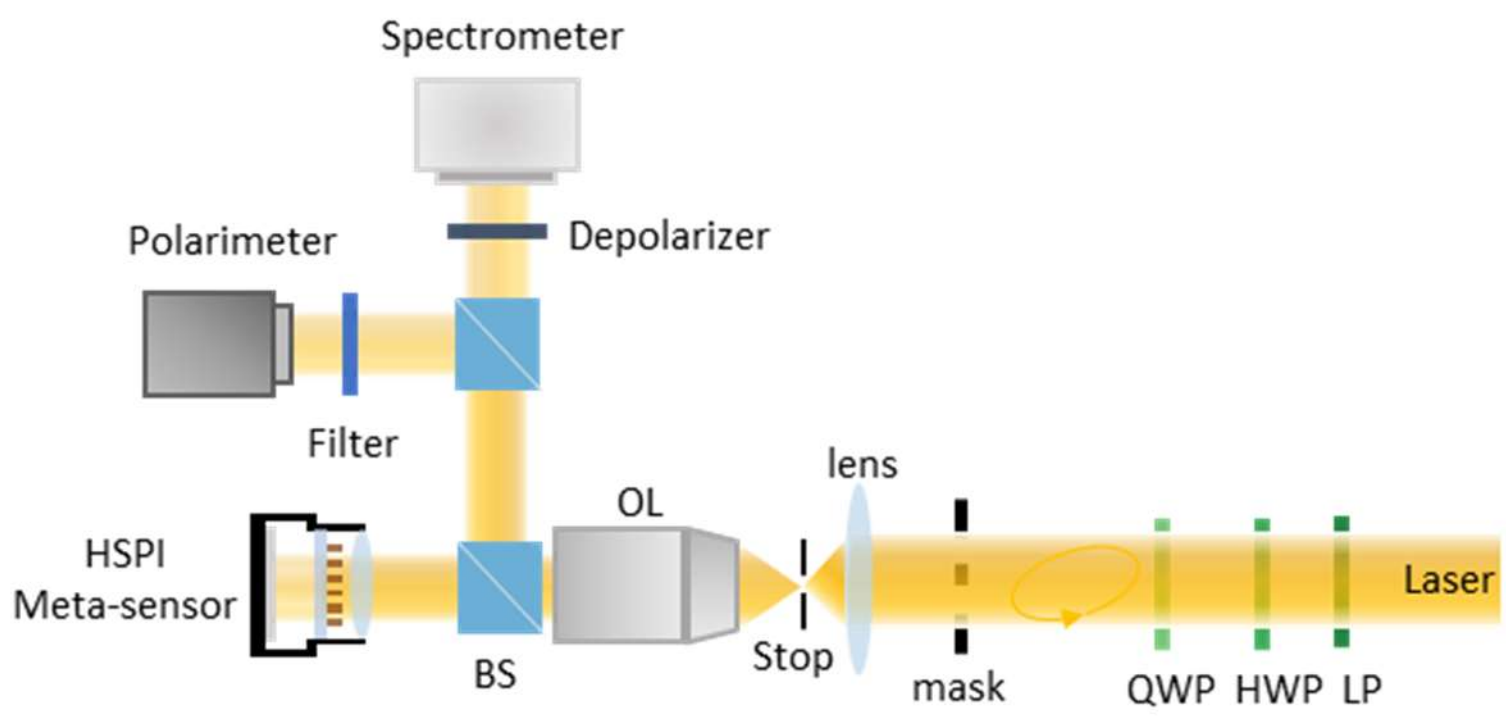

Figure S5 Schematic of the experiment setup. Our designed metasurface is mounted in front of a near-infrared InGaAs FPA (resolution: 512×640, pixel size: 15 μm). An aspheric lens (Thorlabs AL2520) is positioned in front of the metasurface to focus incoming light. Polarization state control is achieved using a linear polarizer (LBTEK FLP25-NIR-M), a half-wave plate (LETEK AHWP110-LNIR), and a quarter-wave plate (LBTEK AQWP10-LNIR). The optical path includes an objective lens (Mitutoyo MY20X-824) and a beam splitter (Thorlabs BSW29R), which divides the light into two paths: one directed to the metasurface-integrated imaging device, and the other to either a polarimeter (Thorlabs PAX1000IR2) or a spectrometer (Avantes NIR) for ground-truth measurements.

Note 7 Central light leakage fabrication imperfections

To address this concern, we conducted a FDTD numerical simulations to clarify the central light leakage under 1300nm.

As illustrated in the SEM images (Figure S6a), the fabricated metasurface exhibits structural deviations from the design, characterized by a distinct tapering shape. This geometric imperfection results in trapezoidal nanopillars with a top-to-bottom width gradient. The top width is approximately 15% smaller than the bottom width. Consistent with our experimental observations, numerical simulations (Figure S6b and c) confirm that this sidewall taper acts as the primary mechanism for the emergence of zeroth-order (on-axis) light leakage.

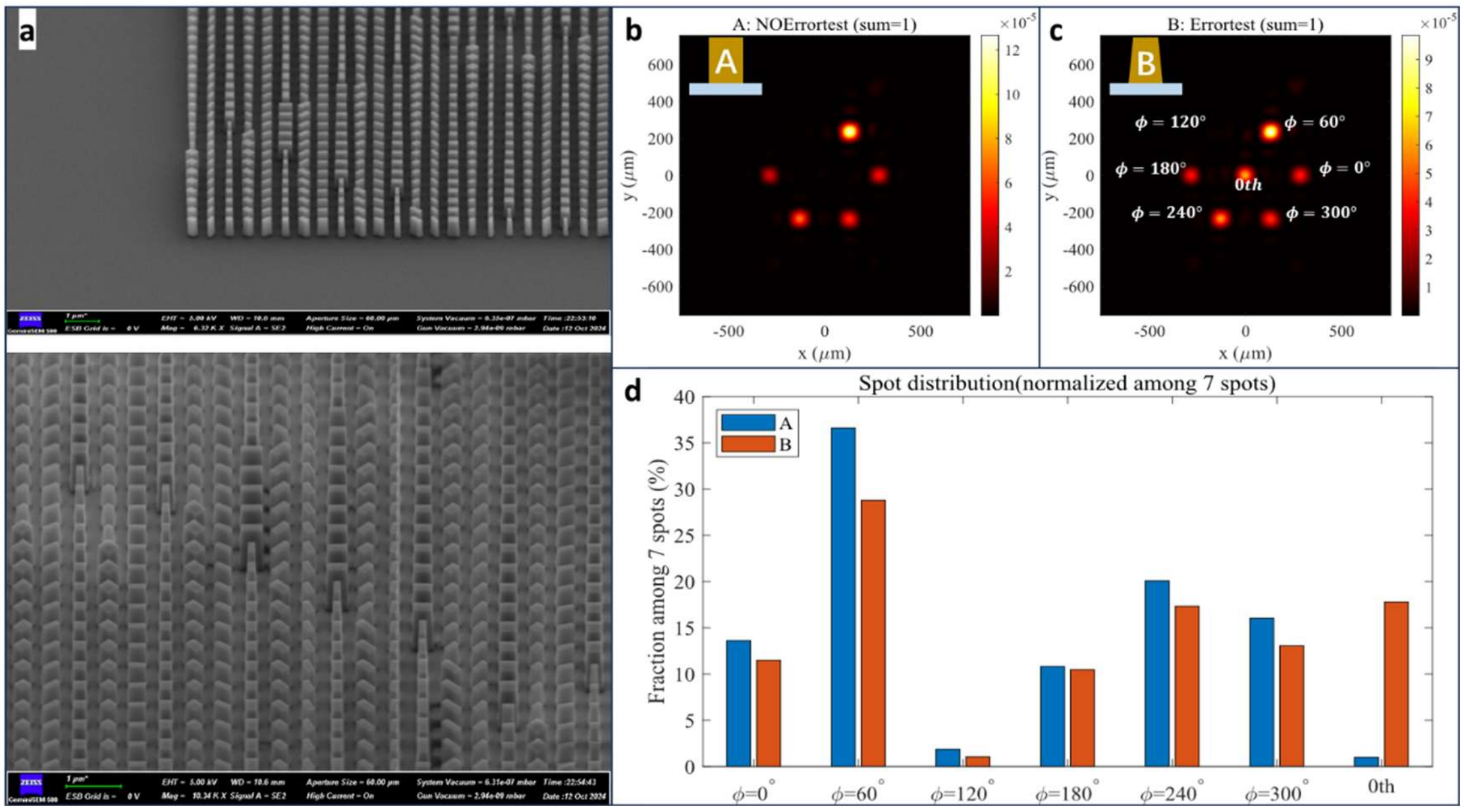

Figure S6 (a) SEM images of the fabricated metasurface. (b) Ideal design with sidewall angle of 90° and its far-field distribution under 1300nm H polarization illumination. (c) Practical fabrication with a ~15% top-to-bottom width reduction and its far-field distribution under 1300nm H polarization illumination. (d) Relative energy distribution between the ideal and practical designs.

Based on the aforementioned physical mechanism of the on-axis leakage, we draw the following deductions regarding the system's performance:

(1) Stability of Diffraction Geometry: The spatial coordinates of the six off-axis diffraction spots remain highly robust. As shown in Figure S6b-c, both the ideal and the tapering nanopillars steer light to the prescribed positions. This suggests that fabrication imperfections do not alter the fundamental phase-gradient of the metasurface, but rather perturb efficiency across different diffraction orders and different channels.

(2) Emergence of the 0th-order Component. The tapering shape significantly breaks the ideal phase modulation, leading to a pronounced on-axis (0th diffraction order) component. While the Fourier-plane center of the ideal design remains nearly dark (Figure S6b), the introduction of a tapering profile results in a conspicuous and intense central spot (labeled "0th" in Figure S6c).

(3) Quantitative Energy Redistribution. To quantify this effect, we performed a power analysis across all seven diffraction spots. As illustrated in Figure S6d, for the ideal case, most of the energy is distributed among the six off-axis polarization channels with

the 0th diffraction order contribution being negligible (1%). Conversely, for the tapering nanopillar, the 0th diffraction order energy increases markedly to 18% of the total diffracted energy. This redistribution makes the central leakage a non-negligible factor that the system must account for during reconstruction

In summary, the impacts of fabrication non-uniformity on the reconstruction performance can be categorized into the following three aspects:

(1) Increased inter-channel crosstalk. The structural deviation perturbs the local amplitude and phase responses of the designed meta-atoms, deviating the polarization-dependent mapping from the design. As a result, the intended diffracted beams are no longer routed with the ideal angular separation, and a non-negligible portion of the optical power is misdirected into 0th diffraction order. Quantitatively, the undesired 0th diffraction order leakage increases from ~1% in the ideal case to ~18% in the nanopillar-tapering case, corresponding to an ~18× increase. Consistently, the total power delivered to the designed six first-order channels decreases from ~99% to ~82%, indicating that ~17% of the energy is redirected into the 0th diffraction order. Moreover, perturbations of the Jones-matrix elements degrade the designed polarization selectivity so that cross-polarized components are partially transmitted/converted and subsequently collected by other channels. This is reflected by channel-wise redistribution (e.g., the $\varphi = 60°$ channel decreases from ~33.8% to ~28.9%). Together, these effects quantitatively confirm increased inter-channel crosstalk and degraded routing fidelity.

(2) Reduced effective SNR and contrast. Fabrication-induced energy reallocation into the 0th diffraction order inevitably compresses the intensity contrast between distinct polarization channels. This effectively diminishes the polarization extinction ratio and channel separability, thereby restricting the dynamic range of discriminative information available for high-fidelity reconstruction. Importantly, the 0th diffraction order should not be regarded as a trivial or uninformative background. Instead, it constitutes a coherent component of the overall system response, which can be constructively leveraged by HSPI-net to enhance information extraction.

(3) Compensation of Systematic Bias. Fabrication imperfections introduce stable, reproducible offsets in amplitude, phase, and polarization response. These perturbations are intrinsically captured during the network training. Within the parameter space covered by the training set, the HSPI-net effectively self-calibrates and compensates

for these stable biases, mitigating their impact on the final output.

Note 8 System field of view (FOV)

The FOV of our system is governed by the interplay between the detector's active area and the metasurface's physical aperture (Figure S7). To rigorously quantify this, we developed a wavelength-dependent FOV model based on the chief-ray trajectory analysis. The effective object-space FOV is determined by the intersection of two primary geometric constraints:

(a) FPA capture constraint: The image point for any wavelength at any polarization channel must fall within the active sensor area to avoid information loss;

(b) metasurface aperture constraint: The chief ray associated with an object-space must remain within the 0.96 mm×0.96 mm metasurface footprint.

We parameterize the object-space FOV using the chief-ray incidence angle $\theta=\left(\theta_x,\theta_y\right)$, where θ_x and θ_y denote the angular deviations from the optical axis in two orthogonal directions. The specific computational steps to determine the FOV are as follows:

Step1: Channel-center displacement (trajectory) on the FPA

For the *i*-th channel, the metasurface introduces a deflection angle $\alpha_i\left(\lambda\right)$ at wavelength λ, with azimuth β_i (for six channels, $\beta_i=0^\circ,60^\circ,\ldots,300^\circ$). The metasurface is placed near the focal plane of Lens2. By accounting for the free-space propagation distance *d*, we convert the wavelength-dependent beam deflection into a measurable lateral coordinate shift (Δr) on the FPA:

$$\Delta r_i\left(\lambda\right)=d\cdot tan\left(\alpha_i\left(\lambda\right)\right) \tag{S18}$$

which can be decomposed into Cartesian components:

$$\begin{aligned}\Delta x_i\left(\lambda\right)&=\Delta r_i\left(\lambda\right)cos\beta_i\\ \Delta y_i\left(\lambda\right)&=\Delta r_i\left(\lambda\right)sin\beta_i\end{aligned} \tag{S19}$$

Accordingly, the channel centers $\left(\Delta x_i\left(\lambda\right),\Delta y_i\left(\lambda\right)\right)$ form wavelength-dependent trajectories on the FPA. When $d\alpha/d\lambda>0$, increasing λ yields a larger $\alpha\left(\lambda\right)$ and

thus a larger $\Delta r(\lambda)$. Therefore, the long-wavelength channel centers move closer to the sensor boundary. This directly explains why the effective FOV becomes more restrictive at the long-wavelength end.

In our calculation, the angular dispersion rates k_i are obtained by fitting experimental data: $k = 0.005824^\circ / \mathrm{nm}$ for the linear-polarization channels and $k = 0.006243^\circ / \mathrm{nm}$ for the circular-polarization channels. Over the operating band, $\alpha_i(\lambda)$ is well approximated by a linear model (Figure S7):

$$\alpha_i(\lambda) = \alpha_{d,i} + k_i(\lambda - \lambda_d) \tag{S20}$$

where $\alpha_{d,i}$ is the deflection angle at the design wavelength λ_d.

Step2: Geometric mapping from object-space angle to the FPA

For an object-space chief-ray angle (θ_x, θ_y), the geometric displacement on the FPA produced by the imaging system (excluding the metasurface dispersion term) can be expressed as

$$\begin{aligned} x_{img} &= f_{\mathrm{eff}} \cdot tan(\theta_x) \\ y_{img} &= f_{\mathrm{eff}} \cdot tan(\theta_y) \end{aligned} \tag{S21}$$

where f_{eff} is the effective focal length of the system. After adding the channel-dependent dispersion-induced shift, the hit position on the FPA for channel i at wavelength λ becomes

$$\begin{aligned} x_{FPA}(\lambda, i) &= f_{\mathrm{eff}} \cdot tan\theta_x + \Delta x_i(\lambda) \\ y_{FPA}(\lambda, i) &= f_{\mathrm{eff}} \cdot tan\theta_y + \Delta y_i(\lambda) \end{aligned} \tag{S22}$$

Step3: Object-space half-angle limit imposed by the FPA capture constraint

Let the active sensor bounds be $|x| \le W/2$ and $|y| \le H/2$, and introduce a safety margin m_{FPA}. To ensure that, at a given wavelength, all channels are fully captured, we require:

$$\begin{aligned} |x_{FPA}(\lambda, i)| &\le W/2 - m_{\mathrm{FPA}} \\ |y_{FPA}(\lambda, i)| &\le H/2 - m_{\mathrm{FPA}} \end{aligned} \tag{S23}$$

Taking the most restrictive channel yields the wavelength-dependent object-space half-

angle limits:

$$\theta_{x,max}^{FPA}(\lambda) = arctan\left(\frac{\frac{W}{2} - \max_i |\Delta x_i(\lambda)| - m_{FPA}}{f_{\mathrm{eff}}}\right)$$
$$\theta_{y,max}^{FPA}(\lambda) = arctan\left(\frac{H/2 - \max_i |\Delta y_i(\lambda)| - m_{FPA}}{f_{\mathrm{eff}}}\right) \quad (S24)$$

These expressions explicitly show that, as λ increases, the outward shift of the channel centers increases $\max_i |\Delta x_i(\lambda)|$ and $\max_i |\Delta y_i(\lambda)|$, thereby shrinking $\theta_{x,max}^{FPA}(\lambda)$ and $\theta_{y,max}^{FPA}(\lambda)$. The long-wavelength end thus imposes the strictest FOV limitation.

Step4: Object-space half-angle limit imposed by the finite metasurface aperture

The metasurface clear aperture is $0.96\,\mathrm{mm} \times 0.96\,\mathrm{mm}$, with half-width $a = 0.48\,\mathrm{mm}$. An object-space angle θ causes a lateral shift of the chief ray (or equivalently, the beam footprint center) on the metasurface plane, which under the paraxial approximation can be written as:

$$x_{ms} = L_{ms} \cdot tan(\theta_x)$$
$$y_{ms} = L_{ms} \cdot tan(\theta_y) \quad (S25)$$

where L_{ms} is the effective distance mapping object-space angle to the metasurface-plane shift. With a metasurface-edge margin m_{MS}, the transmission condition becomes

$$|x_{ms}| \le a - m_{MS}$$
$$|y_{ms}| \le a - m_{MS} \quad (S26)$$

Therefore, the aperture-limited object-space half-angle limits are:

$$\theta_{x,max}^{MS} = arctan\left(\frac{a - m_{MS}}{L_{ms}}\right)$$
$$\theta_{y,max}^{MS} = arctan\left(\frac{a - m_{MS}}{L_{ms}}\right) \quad (S27)$$

Step5: Final FOV as the intersection of the two constraints

Combining the FPA capture constraint and the metasurface-aperture constraint (Figure S7), the wavelength-dependent object-space half-angle FOV is

$$\theta_{x,max}(\lambda) = min\left(\theta_{x,max}^{FPA}(\lambda), \theta_{x,max}^{MS}\right)$$
$$\theta_{y,max}(\lambda) = min\left(\theta_{y,max}^{FPA}(\lambda), \theta_{y,max}^{MS}\right) \quad (S28)$$

Accordingly, the wavelength-dependent full FOV in x and y is

$$FOV_x = 2\theta_{x,max}(\lambda)$$
$$FOV_y = 2\theta_{y,max}(\lambda) \quad (S29)$$

Step6: Diagonal FOV

To give a diagonal object-space field of view, we define the diagonal half-angle as the corner-diagonal of the rectangular angular support determined by the horizontal and vertical half-angles. Specifically, after applying the FPA-capture and metasurface-aperture constraints, we obtain wavelength-dependent object-space half-angles $\theta_{x,max}(\lambda)$ and $\theta_{y,max}(\lambda)$ along two orthogonal directions.

Instead of combining angles directly, we combine them in the $tan(\cdot)$ domain because, under the paraxial imaging geometry, object-space angles map to lateral displacements on the image plane via:

$$x_{img} = f_{\text{eff}} \cdot tan\left(\theta_{x,max}(\lambda)\right)$$
$$y_{img} = f_{\text{eff}} \cdot tan\left(\theta_{y,max}(\lambda)\right) \quad (S30)$$

Therefore, the "diagonal" extent corresponds to the radial displacement of the corner point $(x_{max}, y_{max}) = \left(f_{\text{eff}} \cdot tan\left(\theta_{x,max}(\lambda)\right), f_{\text{eff}} \cdot tan\left(\theta_{y,max}(\lambda)\right)\right)$, yielding:

$$r_{max}(\lambda) = \sqrt{x_{max}^2 + y_{max}^2} = f_{\text{eff}} \cdot \sqrt{\left(tan\left(\theta_{x,max}(\lambda)\right)\right)^2 + \left(tan\left(\theta_{y,max}(\lambda)\right)\right)^2} \quad (S31)$$

We then convert this radial displacement back to an equivalent diagonal half-angle:

$$\theta_{d,max}(\lambda) = \arctan\left(\frac{r_{max}(\lambda)}{f_{\text{eff}}}\right) = \arctan\left(\sqrt{\left(tan\left(\theta_{x,max}(\lambda)\right)\right)^2 + \left(tan\left(\theta_{y,max}(\lambda)\right)\right)^2}\right) (S32)$$

Accordingly, the diagonal full FOV is defined as:

$$FOV_{dia} = 2\theta_{d,max}(\lambda) \quad (S33)$$

while the horizontal and vertical full FOVs are $FOV_x = 2\theta_{x,max}(\lambda)$ and $FOV_y = 2\theta_{y,max}(\lambda)$. This definition is consistent with the rectangular sensor geometry and the angle-to-displacement mapping, and it avoids the inaccuracy that would arise from directly combining angles via $\sqrt{\theta_x^2 + \theta_y^2}$ when the $tan(\cdot)$ nonlinearity is non-

negligible.

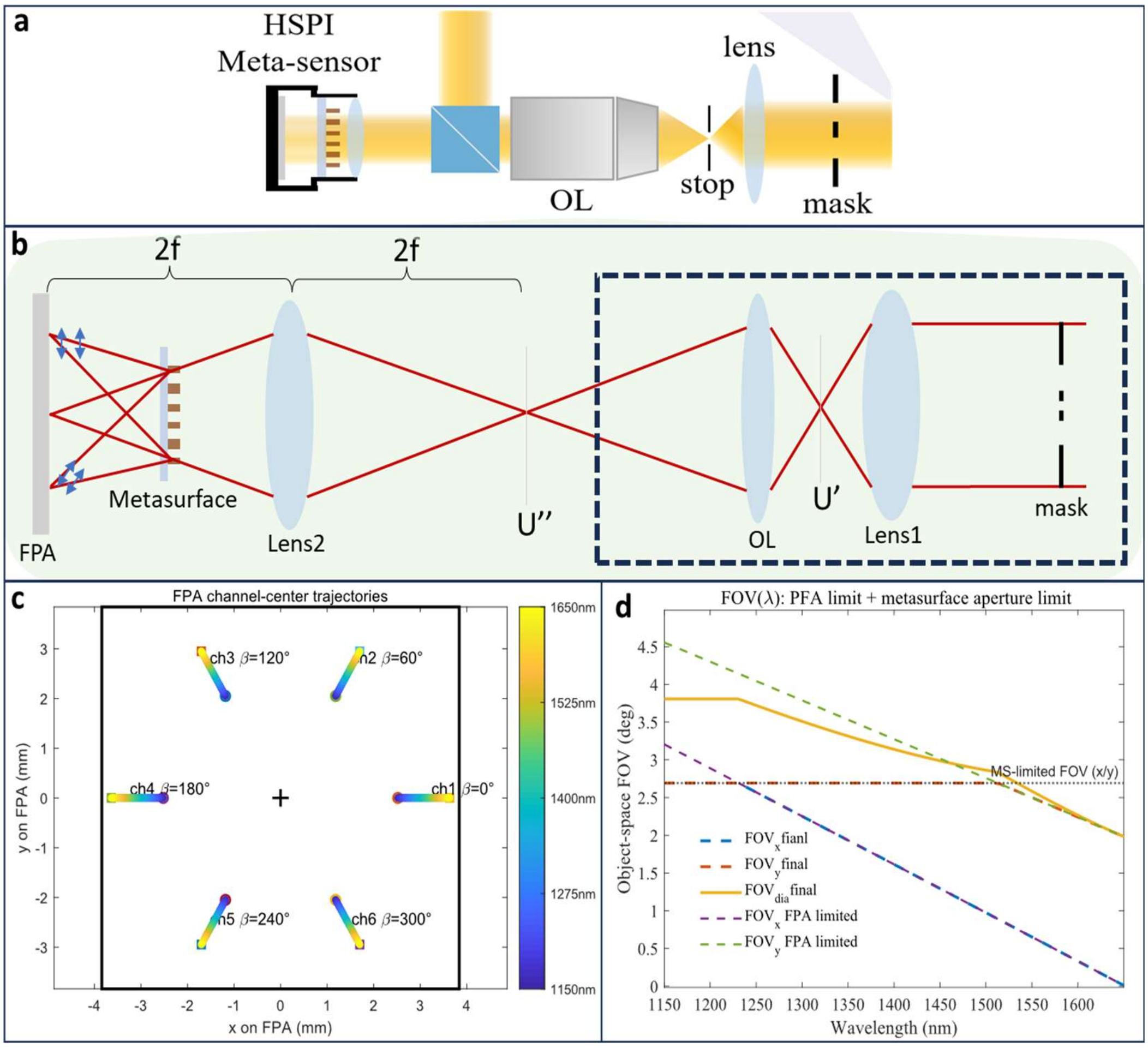

Figure S7(a-b) Schematic illustration of the system imaging-process analysis. (c) Projected spectral dispersion footprint on the focal plane array (FPA). (d) Wavelength-dependent FOV constraints imposed by the FPA and the metasurface aperture; blue and orange curves represent the final FOV_x and FOV_y, respectively. yellow curve denotes the final diagonal FOV.

Note 9 Imaging spatial resolution

We performed additional experimental characterization using a standard USAF 1951 resolution target. Under the 1300 nm operating wavelength, the system demonstrates a spatial resolving capability of 10.07 lp/mm.

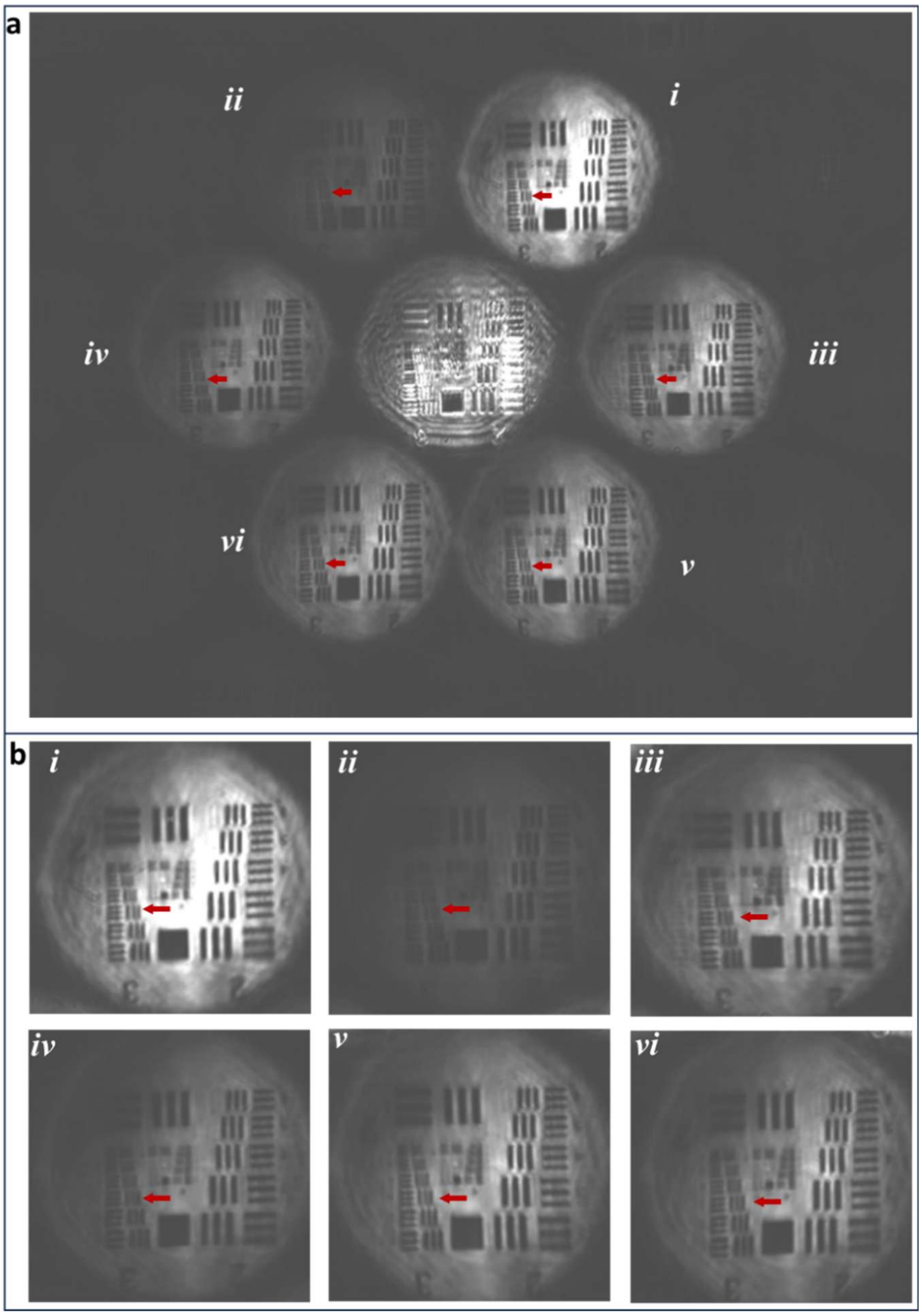

Figure S8 USAF 1951 resolution target test

Note 10 Metasurface efficiency

To provide a physically meaningful metric for a computational imaging system, we defined η_{total} as the ratio between the total power collected by the FPA across the seven active channels (i.e. CH0 ~ CH6) to the incident power within the metasurface clear aperture:

$$\eta_{total}(\lambda) = \frac{\sum_{o=0}^{6} P_o(\lambda)}{P_{in}(\lambda)} \quad (S34)$$

This definition focuses on the usable photon flux available for encoding and detection, which is more relevant to the system's SNR than the intrinsic material transmittance. We measured η_{total} from 1.15 to 1.65 μm with a 50 nm increment. As shown in Figure S9, η_{total} varies slowly across the spectrum, remaining between 19% and 37%, with a broadband average of 30%. This indicates that the system maintains a substantial throughput sufficient for high-quality imaging.

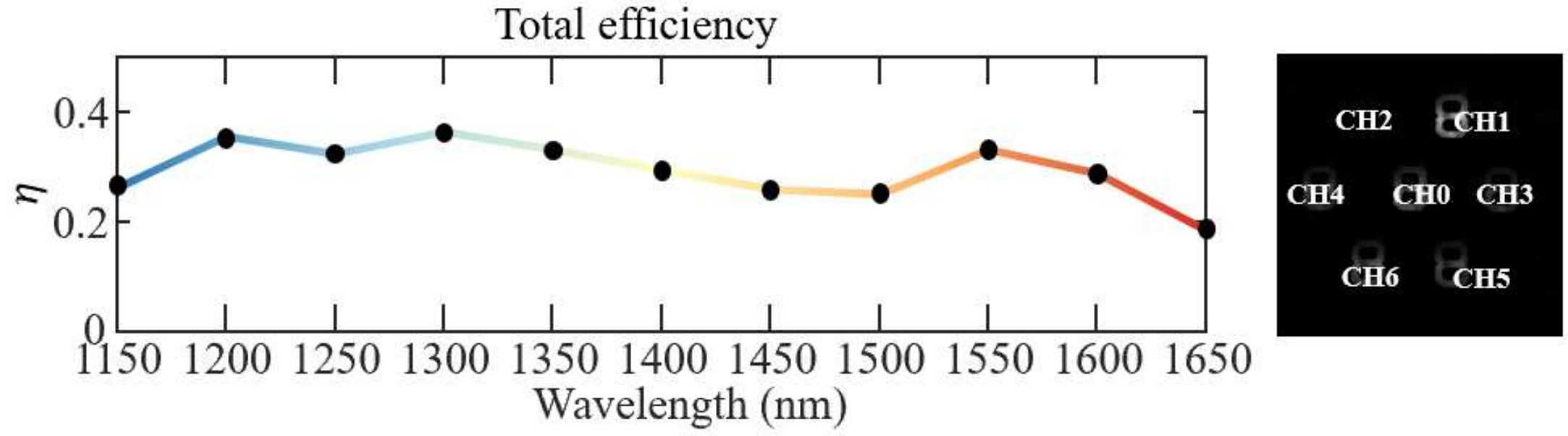

Figure S9 Measured metasurface efficiency

Note 11 Implement of the deep learning

At a fixed laser wavelength of 1.3 μm, we adjusted the relative positions of the half-wave plate (HWP) and quarter-wave plate (QWP) in the test optical path to ensure that the collected polarization states uniformly covered the entire Poincaré sphere, with a resolution of 1°. The polarization states measured by a commercial polarization analyzer were used as ground truth. In total, 8281 polarization states were collected to construct 8281 data samples. Among them, 182 samples were selected for network prediction, while the remaining 8099 were used for training. The training and validation sets were split at a ratio of 0.8:0.2. The training process is detailed in Figure S4a.
Under a fixed polarization state, a continuously tunable supercontinuum laser was used to input laser light with wavelengths ranging from 1.15 to 1.65 μm in 0.1 nm steps. The spectra measured by a commercial spectrometer served as ground truth. A total of 5001 spectral samples were collected. 9 samples spaced at 50 nm intervals were selected for prediction, and the remaining 4992 were used for training. The training/validation split was 0.8:0.2. The training process is shown in Figure S4b.

Under a fixed polarization state, a continuously tunable supercontinuum laser and a single-frequency 1.3 μm laser were used. The supercontinuum laser provided inputs from 1.15 to 1.65 μm with 0.2 nm step size to construct bimodal spectra. The ground truth was obtained from a commercial spectrometer. A total of 2498 spectral samples were collected. 50 samples around 1.3 μm were selected for prediction, and the remaining 2448 were used for training, with a training/validation split of 0.8:0.2. The training process is illustrated in Figure S4c.

Under a fixed polarization state, a multi-channel output from a supercontinuum laser was used. The ground truth spectra were measured by a commercial spectrometer. Within the wavelength range of 1.15–1.65 μm, 22815 spectral combinations were constructed through permutation. Of these, 2285 samples were used for prediction, and the remaining 20530 samples were used for training, with a training/validation split of 0.8:0.2. The training process is shown in Figure S4d.

Using the test system illustrated in Fig. 3a (main text), laser beams at 1.18 μm and 1.3 μm from a supercontinuum laser were input. The polarization states and spectra measured by a commercial polarization analyzer and spectrometer served as ground truth. By independently tuning the polarization states of the two wavelengths using wave plates, 270323 polarization-spectral combinations were constructed. Among these, 2000 samples were used for prediction, and the remaining 268323 were used for training. The training/validation split was 0.8:0.2. The training process is shown in Figure S4e.

Under a fixed polarization state, we collected 78 LVF-based hyperspectral samples: 76 were used to train the HSPI-net, using a training/validation split of 0.8:0.2. And the remaining 2 samples were used for inference. The training process is detailed in Figure S4f.

At a fixed laser spectral center wavelength of 1.2 μm,1.13μm,1.48μm and 1.6μm, we again adjusted the relative positions of the HWP and QWP to uniformly cover the entire Poincaré sphere with a resolution of 5°. The polarization states and corresponding spectra measured by commercial polarization and spectral analyzers served as ground truth. 199470 samples were collected. Among these, 4802 samples were used for network prediction, while the remaining 194688 samples were used for training, using a training/validation split of 0.8:0.2. The training process is detailed in Figure S4g.

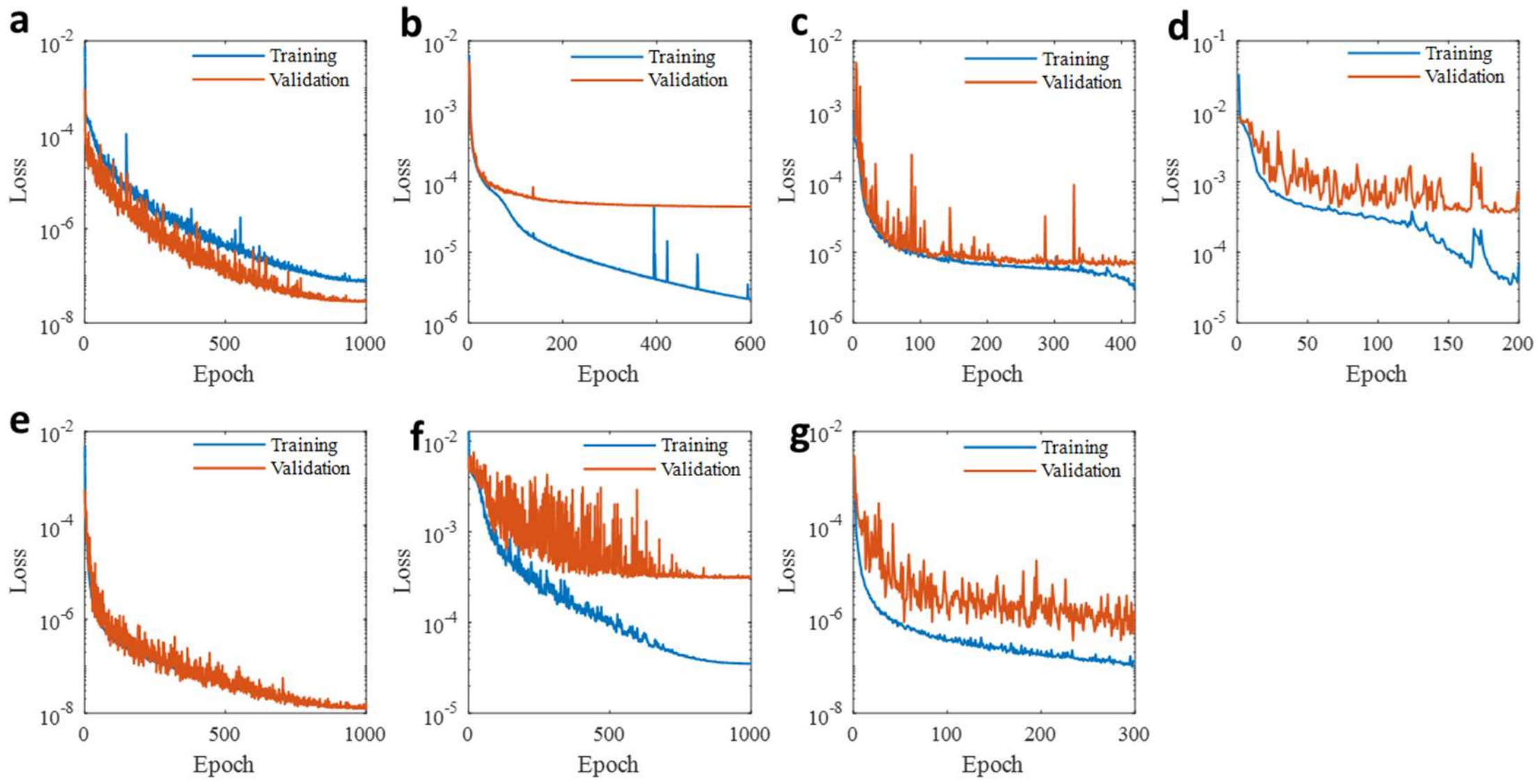

Figure S10. Training and validation loss curves during the network training. (a)Training process of the full-Stokes polarization state reconstruction network (Fig.3c in main text). (b) Training process of the narrowband spectral reconstruction network (Fig.3f in main text). (c) Training process of the double-peak spectral reconstruction network (Fig.3g in main text). (d) Training process of the multi-peak spectral reconstruction network ((Fig.3h in main text)). (e) Training process of the polarization-dependent spectral reconstruction network (Fig.4 in main text). (f) Training process of the hyperspectral polarization imaging (HSPI) (Fig.5b-e in main text). (g) Training process of the hyperspectral polarization imaging (HSPI) (Fig.5f in main text).

Note 12 Extended reconstruction results

Extended reconstruction results of complex spectral and hyperspectro-polarimetric imaging are presented in Figure S11, Figure S12 and Figure S13. Additionally, statistical analyses of the error distributions corresponding to these results are provided. The average reconstruction error, measured by the L1 norm, is 30.22 %, while the average root mean square error (RMSE) is 2.32 %. The L1 norm is defined as:

$$\varepsilon_{L1} = \frac{\| I_0 - I_R \|_1}{\| I_0 \|_1} \tag{S35}$$

Where I0 denotes the ground truth spectrum and IR the reconstructed spectrum.

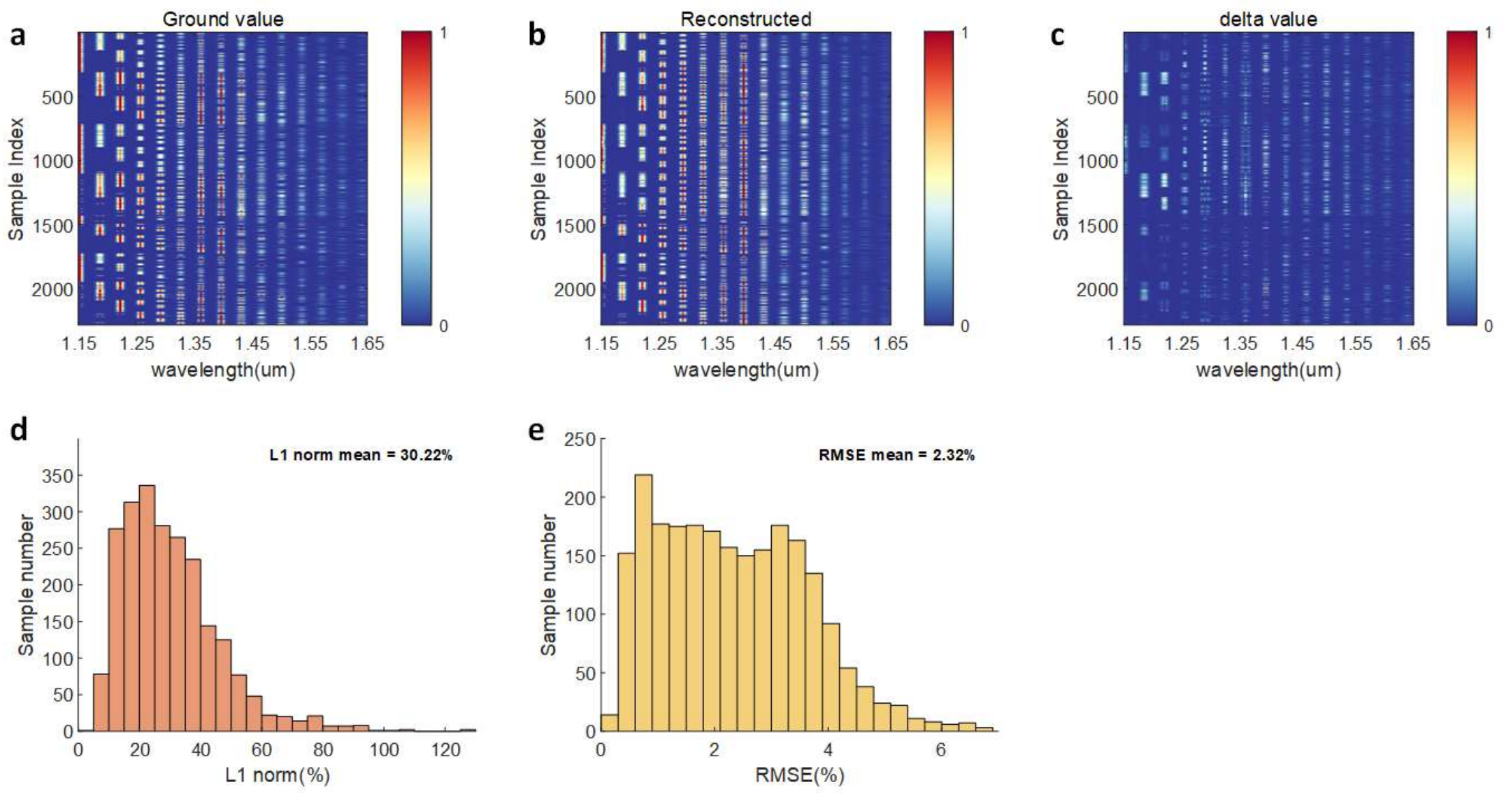

Figure S11. A total of 2285 samples for the spectral reconstruction. (a) Ground-truth spectra. (b) Reconstructed spectra. (c) Absolute error between ground truth and reconstructed spectra. (d-e) Statistical histograms of L1 norm and RMSE across all 2285 samples, comparing reconstructed results with the ground truth.

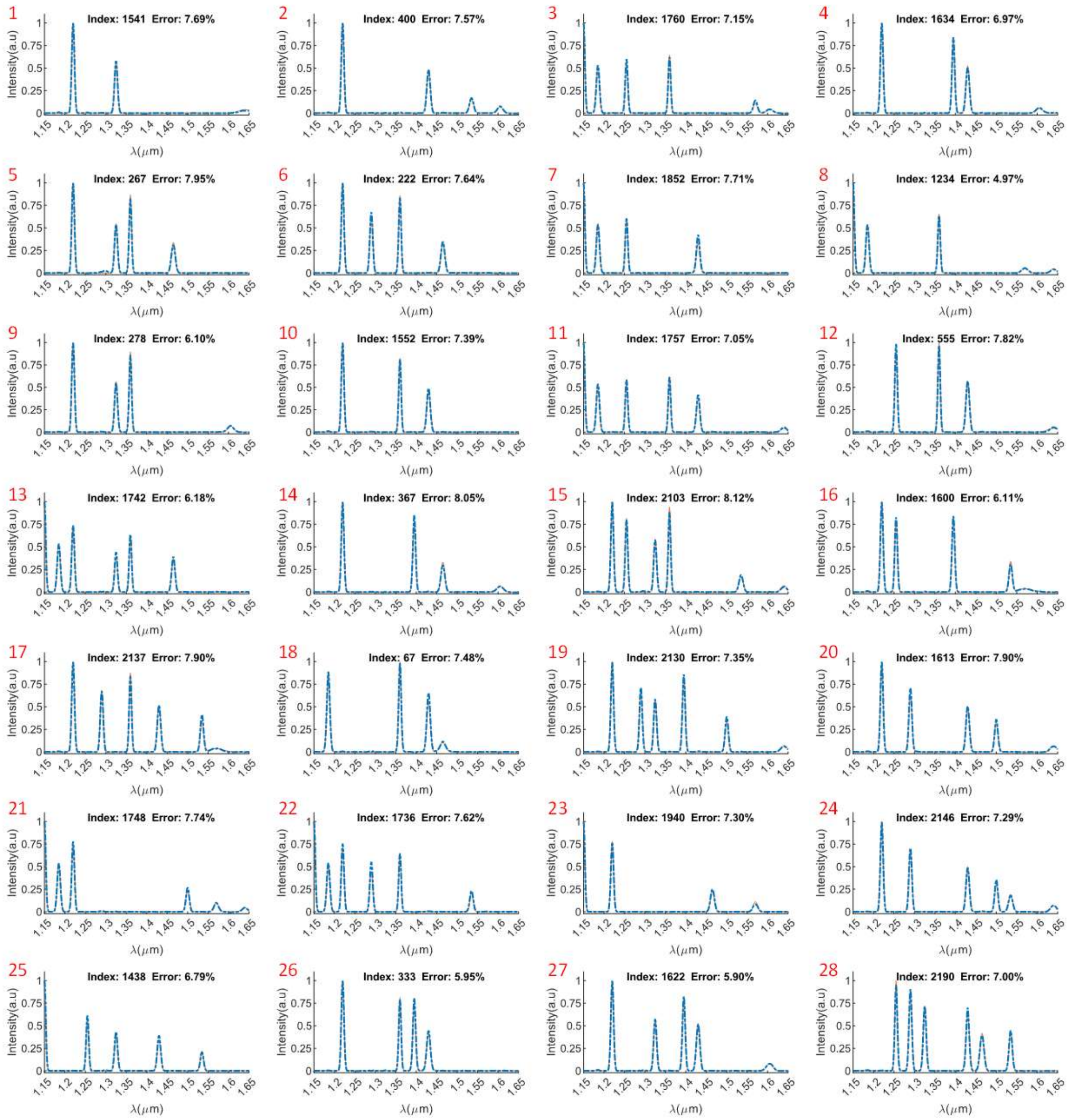

Figure S12 Spectral reconstruction results for 28 randomly selected samples from a dataset of 2285. 'Index' denotes the sample identifier, while 'Error' corresponds to the L1 norm.

Peak Signal-to-Noise Ratio (PSNR) and Structural Similarity Index (SSIM) are used to evaluate the accuracy of the reconstruction target. They are defined as:

$$PSNR = 10\log_{10}\left(\frac{Max_{val}^{\ 2}}{\frac{1}{N}\sum_{i=1}^{N}\left(x'_i - x_i\right)^2}\right) \tag{S36}$$

$$SSIM(x, x') = \frac{(2\mu_x\mu_{x'})(2\sigma_{xx'})}{(\mu_x^{\ 2} + \mu_{x'}^{\ 2})(\sigma_x^{\ 2} + \sigma_{x'}^{\ 2})} \tag{S37}$$

For the PSNR, the maximum value (Maxval) is set to 1, where x represents the reconstructed values and x' represents the ground truth values. The SSIM between x and x' is defined as SSIM(x, x'), where μ_x,$\mu_{x'}$ represent the mean values of x and x' ,σ_x^2,$\sigma_{x'}^2$ represent the variances of x and x',$\sigma_{xx'}$ represent the covariance between x and x' .

The polarization states employed for HSPI reconstruction are shown in Figure S13a. Spectral center wavelengths of 1.2 µm,1.13µm,1.48µm and 1.6 µm share the same polarization state, resulting in a total of 4802 wavelength-polarization combined states. The HSPI exhibits an average reconstruction error with an RMSE of 0.17 %, a PSNR of 55.67 dB, an L1 norm of 2.74 %, and an SSIM of 99.77 %.

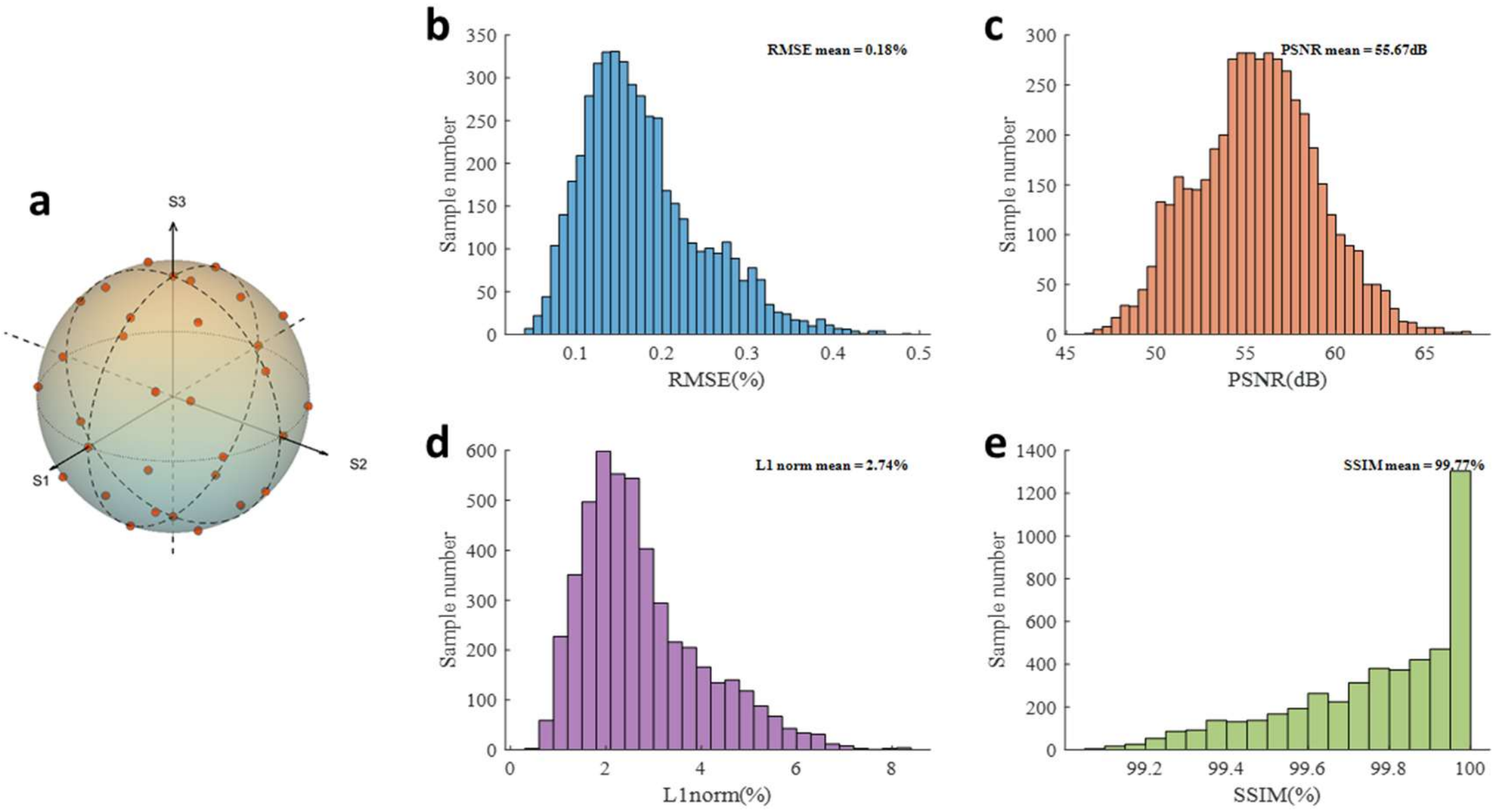

Figure S13 HSPI reconstruction results of polarization and imaging for 4802 randomly selected samples. (a) Selected 1.2um (49 states),1.13um (49 states),1.48um (49 states) and 1.6um (49 states) Polarization states visualized on the Poincaré sphere, 4802 wavelength-dependent polarization states were combined from their various polarization states. (b-f) Statistical evaluation of HSPI reconstruction accuracy using RMSE (Root Mean Square Error), PSNR (Peak Signal-to-Noise Ratio), L1 norm, and SSIM (Structural Similarity Index Measure) metrics.

Note 13 Sparse sampling dataset

We conducted a dedicated comparative study by varying the sampling density of the polarization states in the training set. While maintaining the optical system

configuration and the data pipeline, we trained two separate modes with same architecture: one with a dense 1° sampling on the Poincaré sphere and the other with a sparser 2° sampling. Both models were then evaluated using an identical test set and error-analysis protocol to observe the performance degradation.

The results, summarized in Figure S14, demonstrate that HSPI-net maintains remarkable stability even when the training data density is halved.

Figure S14a shows the dense training with 1° sampling. The reconstructed points (orange) overlap precisely with the ground truth (open circles) on the Poincaré sphere. The polarization angle error (ε_p) remains at a low level of 0.134°. Meanwhile, the Stokes-component error distributions are tightly concentrated, with the 3σ bounds of ΔS_1, ΔS_2, ΔS_3 being ±0.008, ±0.010 and ±0.005 (as indicated by the dashed limits in the figure).

Figure S14b shows the sparse training with 2° sampling. Despite the sparser coverage, the reconstructed point follows the ground-truth trajectory with no structural deviation. The polarization angle error (ε_p) only slightly increases to 0.182°, and 3σ ranges for ΔS_1, ΔS_2, ΔS_3 remains ±0.022, ±0.024 and ±0.009.

The marginal increase in error (only ~0.05°) under sparse training confirms that HSPI-net does not merely "memorize" discrete points but effectively learns the underlying continuous physical mapping of the metasurface. Because the metasurface's phase and amplitude modulation vary smoothly across the Poincaré sphere, the network exhibits powerful generalization capability, ensuring that even states sparsely represented during training can be reconstructed with high fidelity.

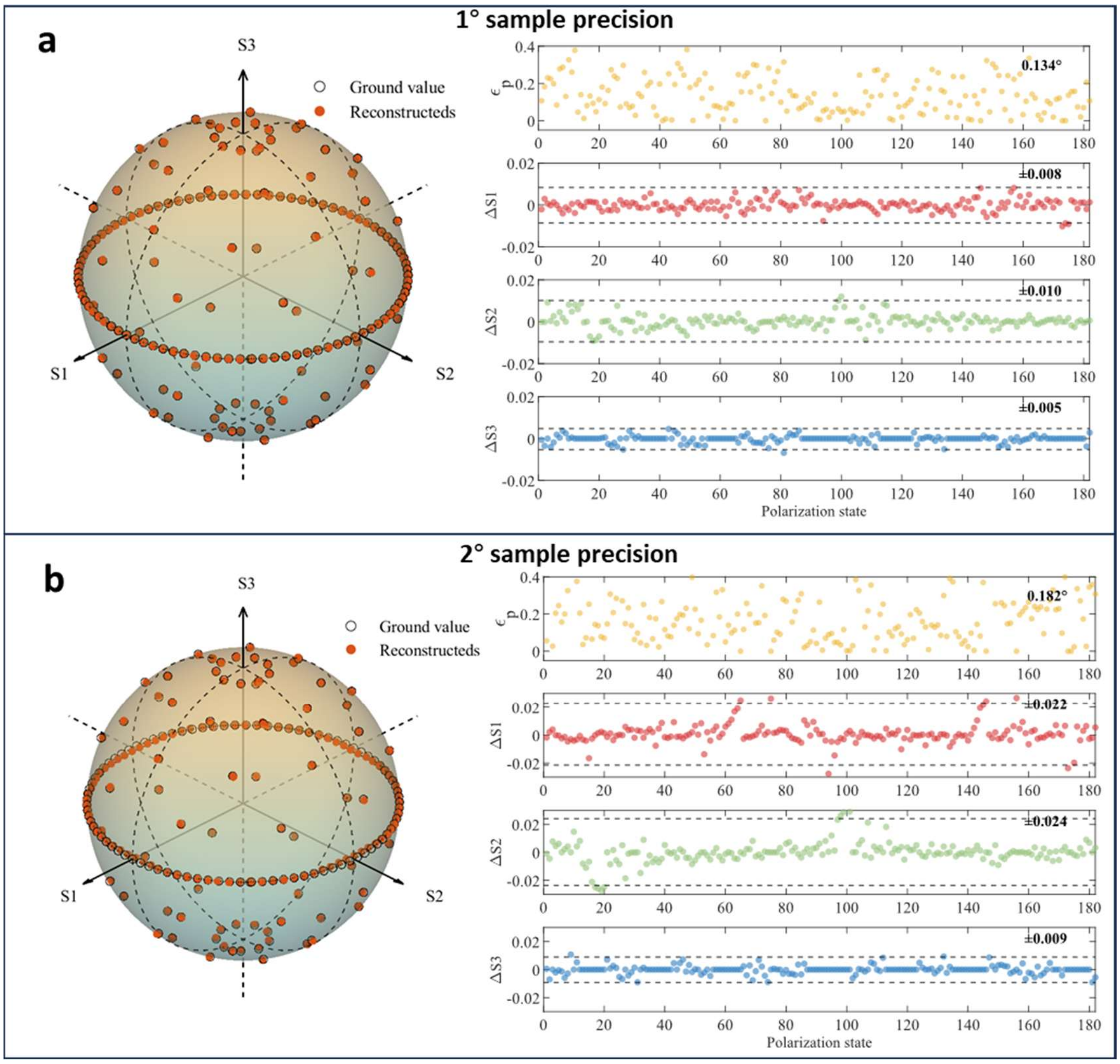

Figure S14 (a) Density training with 1° sampling. (b) Sparse training with 2° sampling. Reconstructed polarization states (orange) and ground truth (open circles) on the Poincaré sphere. Mean angular error ε_p and absolute error distribution of S_i.

Note 14 Generalization capability

The generalization capability of HSPI-net to spectral-polarization distributions beyond those presented in the training set. In our previous demonstrated experiment, we have adopted a strict and fully independent train/test split. It means that the test samples were never involved in parameter updates during training, nor were they exposed to the model through any form of data leakage. Therefore, the reported test performance has reflected our model's generalization capability on previously unseen data. Further, we provided quantitative similarity-based error analysis on the datasets, to completely assess the generalization capability.

To quantify the "closeness" between unseen test samples and the training samples, we adopted a sample-to-sample similarity metric based on the well-established nearest-neighbor search principle. Each spectral-polarization image sample is represented as a high-dimensional vector $x \in \mathbb{R}^d$ (d = 128× 128× 501×4). For any test sample $\boldsymbol{x}_{test}$, we calculate its similarity to the training set $\{\boldsymbol{y}_{test}\}$ using the nearest-neighbor similarity score: $Score(\boldsymbol{x}) = \max_{j\in\{1,\dots,N\}} \{\mathrm{CosSim}(\boldsymbol{x}, \boldsymbol{y}_j)\}$, where 'CosSim' represents the cosine similarity defined by $\mathrm{CosSim}(\boldsymbol{x}, \boldsymbol{y}) = (\boldsymbol{x}^T \boldsymbol{y}) / (\|\boldsymbol{x}\|_2 \|\boldsymbol{y}\|_2)$ (the numerator is the Euclidean inner product, and the denominator is normalizes by the L2 magnitudes). This score quantifies the degree to which a test sample resembles its closest counterpart in the training set. A score approaching 1 indicates that the test sample closely aligns with the statistical patterns captured during training.

As shown in Figure S15, the reconstruction error (RMSE) exhibits a clear correlation with this similarity score. HSPI-net maintains a lower and stable reconstruction error when samples are within high-score distribution. Crucially, the reconstruction error increases gradually rather than catastrophically as similarity decreases, indicating that the network has learned the underlying continuous physical mapping of the metasurface rather than simply memorizing the training labels. Because the metasurface's structural dispersion and phase response are physically continuous functions of wavelength and polarization, the network can effectively interpolate between trained grid points to accurately reconstruct unseen intermediate states.

To further improve generalization against experimental fluctuations, we incorporated multiple perturbations and data augmentations (e.g., varying noise levels) during training. This strategy enhances the stability and robustness of the model on unseen samples. This ensures the model remains robust even when the measured "unseen" distributions are perturbed by environmental noise.

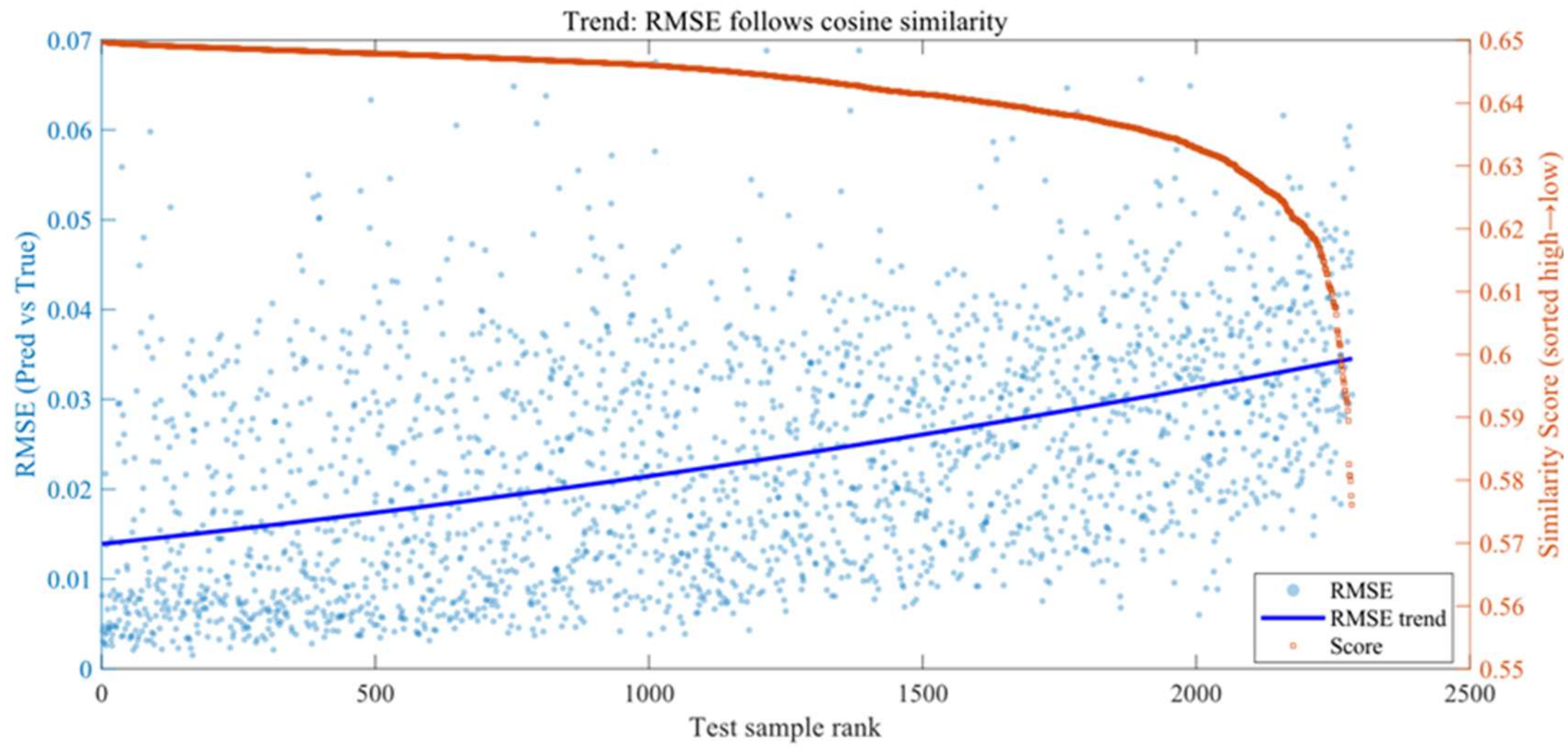

Figure S15 Correlation between reconstruction error and Cosine similarity.

Note 15 HSPI-net for compensating for device errors

During the supervised learning process, HSPI-net treats these non-ideal factors not as random noise, but as deterministic components of the system's transfer function. For stable perturbations (such as the central zeroth-order leakage, quasi-stationary crosstalk, and slowly varying amplitude non-uniformities), the network effectively performs an implicit inverse-mapping. This allows the model to learn a self-calibration mechanism that suppresses systematic errors during reconstruction.

To quantitatively evaluate the compensation efficacy, we performed a comprehensive statistical analysis comparing the conventional Stokes parameters retrieval method with the HSPI-net approach. In retrieving the Stokes parameters using the conventional method, we first perform pixel-level registration of the images acquired from the six channels (H, V, D, A, R, and L). After alignment, the Stokes parameters were directly reconstructed by their definitions: $S_0 = I_H + I_V$, $S_1 = I_H - I_V$, $S_2 = I_D - I_A$, $S_3 = I_L + I_R$, where I_H, I_V, I_A, I_D, I_R and I_L denote the intensities of the six polarization channels, respectively. We visualized the reconstruction errors across approximately 16753 pixels (Figure S16a). For a fixed input state, we calculated the pixel-wise angular deviation between the recovered normalized Stokes vectors and the ground truth. The mean polarization angle errors (ε_p) is 8.00°. Moreover, statistical analysis of the Stokes-component error distributions across all test samples shows that the deviations

of ΔS_1, ΔS_2, and ΔS_3 are tightly confined within the $\pm 3\sigma$ intervals of ± 0.358, ± 0.353, and ± 0.296, respectively.

As an ideal-reference upper bound for the conventional analytical retrieval (assuming an error-free device without fabrication deviations), we use the FDTD-based electromagnetic simulation results of the metasurface under the same incident polarization states. The mean polarization angle errors (ε_p) is 4.37°. With fabrication-induced non-idealities lead to a noticeably degraded analytical retrieval accuracy, yielding an approximately 2-fold larger mean ε_p compared with the ideal FDTD reference.

In contrast, by leveraging the HSPI-net, we evaluated the polarization reconstruction over 182 independent test samples. The average angular error (ε_p) is significantly reduced to only 0.134° . This represents a 59-fold improvement in precision compared to the analytical baseline, demonstrating the network's powerful end-to-end correction of system imperfections. Moreover, statistical analysis of the Stokes-component error distributions across all test samples shows that the deviations of ΔS_1, ΔS_2, and ΔS_3 are tightly confined within the $\pm 3\sigma$ intervals of ± 0.008, ± 0.010, and ± 0.005, respectively Figure S16b). These results confirm that HSPI-net not only minimizes the global reconstruction error but also effectively suppresses systematic biases and fluctuations, ensuring high stability and consistency.

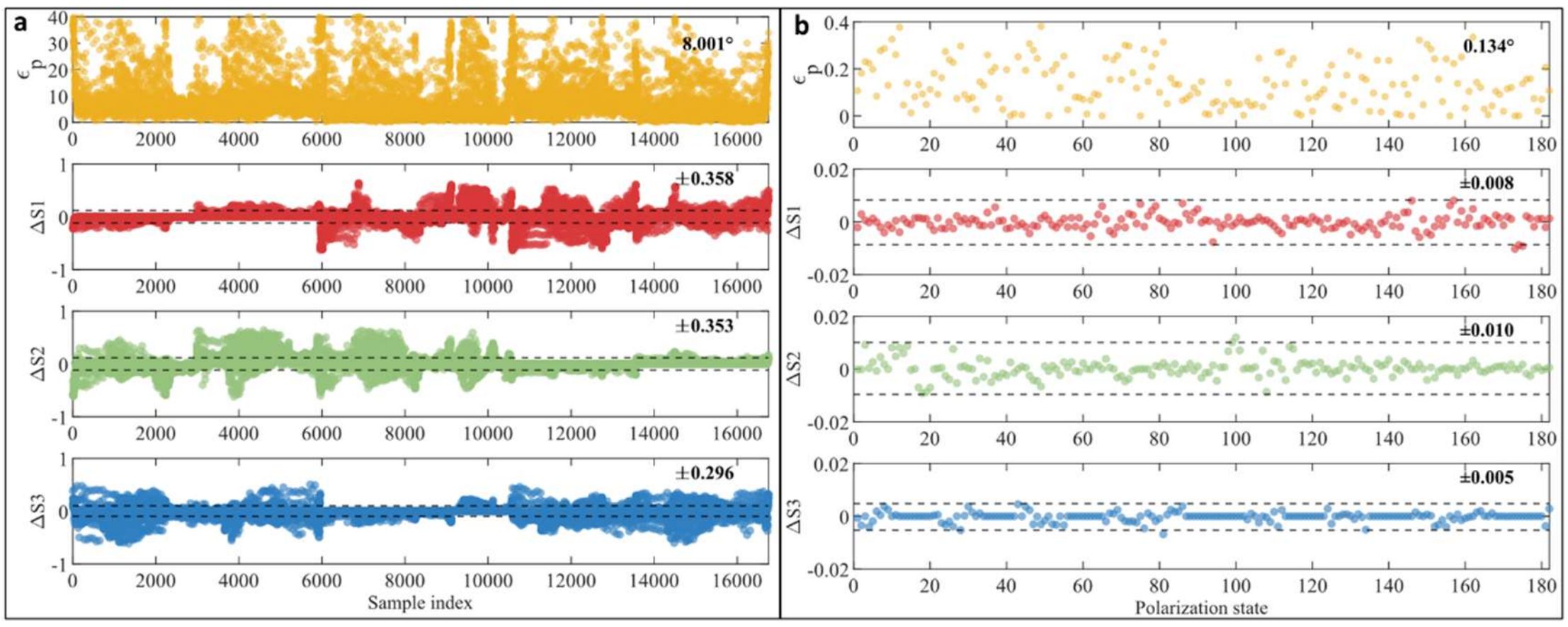

Figure S16 Stokes vectors reconstruction. (a) Using Stokes standard definition. A total of 16753 pixels samples are collected under the six nominal polarization channels (H, V, R, L, D, and A). Quantify the reconstruction error across polarization states: the polarization angular error ε_p on the Poincaré sphere (top), and the component-wise

Stokes deviations ΔS_1, ΔS_2, and ΔS_3. (b) Using the HSPI-net. Quantify the reconstruction error across polarization states: the polarization angular error $\varepsilon_p$on the Poincaré sphere (top), and the component-wise Stokes deviations ΔS_1, ΔS_2, and ΔS_3.

Note 16 Cross-platform deployment and on-device validation on an embedded system

we migrated our original PyTorch inference pipeline to ONNX Runtime, enabling end-to-end deployment on an ARM-based embedded platform. As shownin Figure S17a and b, we exported the HSPI-net to an ONNX computational graph and implemented graph-level optimizations (e.g., operator fusion and constant folding). Furthermore, we explored INT8 quantization (model_quant.onnx) to significantly reduce memory bandwidth requirements and computational overhead, moving the system closer to a practical mobile-level implementation.

We select the Raspberry Pi 5 platform (Quad-core Arm Cortex-A76 @ 2.4GHz; GPU: VideoCore VII) as a proxy for a mobile-level processor. Its CPU performance (~618 single-core / ~1610 multi-core from Geekbench) is representative of mid-tier mobile chipsets around 2021.

The experimental setup, shown in Figure S17c, demonstrates the compact integration of the metasurface camera with the embedded processor. Importantly, this validation does not rely on an external desktop-class NVIDIA GPU; instead, it is completed under mobile/edge-class compute constraints, aligning with the “resource-constrained, mobile-level processor” situation.

As evidenced by the terminal output in Figure S17e, the model successfully executes a full forward pass on the Raspberry Pi 5, producing a complete output tensor of size (1, 2004, 128, 128). Under current unoptimized settings on the Raspberry Pi 5, the mean inference time is ~11.43 s per frame (Figure S17f). This represents an “on-device” execution proof-of-concept. These results provide experimental evidence that HSPI-net is fundamentally deployable on mobile-class hardware. This validates the potential of our method to transition from a laboratory setup to a functional, real-time, compact imaging system.

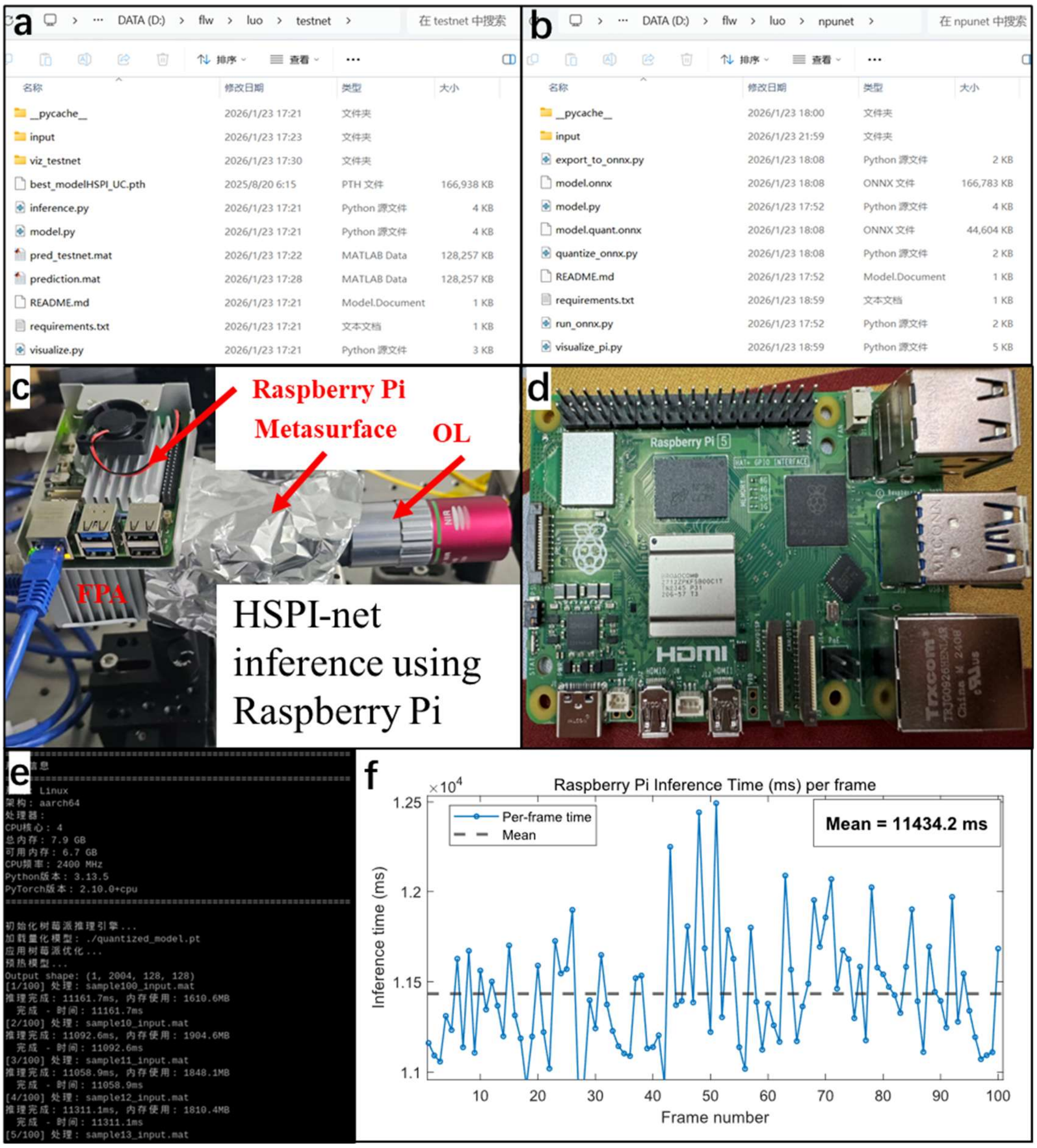

Figure S17 Cross-platform deployment and on-device validation on an embedded system. (a) File structure of the original PyTorch implementation on a desktop environment. (b) Optimized ONNX Runtime deployment structure tailored for ARM-based processor, featuring the INT8-quantized model. (c) Experimental setup illustrating the integration of the metasurface camera with a Raspberry Pi 5. (d) The image of the Raspberry Pi 5 single-board computer. (e) Terminal output demonstrating the successful end-to-end inference of HSPI-net on the Raspberry Pi 5 platform.
